# Phosphorus-dependent shifts in acquisition strategies revealed by integrated transcriptomics and metabolomics in soybean roots

**DOI:** 10.1186/s12870-025-07957-x

**Published:** 2025-12-17

**Authors:** Guangyao Zhao, Tongli Yang, Zhu Chen, Wanping Zhang

**Affiliations:** 1 College of Agriculture, Guizhou University, Guiyang, Guizhou 550025 China; 2 Rongjiang County Tashi Yao and Shui Ethnic Townships Agricultural and Rural Comprehensive Service Center, Qiandongnan, Guizhou 557204 China

**Keywords:** Phosphorus acquisition strategy, Phosphorus gradient, Soybean, Metabolomics, Transcriptomics

## Abstract

Under phosphorus (P) deficiency, soybean (*Glycine max* L.) adapts by modifying root architecture, increasing the release of organic exudates, and engaging arbuscular mycorrhizal (AM) symbiosis. However, how these strategies trade off across a P gradient remains unclear. In this study, we integrated transcriptomic and metabolomic analyses to examine five soybean cultivars under soil P supplies of 0 mg P kg⁻¹ (severe deficiency, P0), 30 mg P kg⁻¹ (moderate deficiency, P30), 60 mg P kg⁻¹ (mild deficiency, P60), 90 mg P kg⁻¹ (adequate, P90), and 120 mg P kg⁻¹ (excess, P120). Our results indicate that plant-available P is associated with shifts among three P acquisition strategies in soybean. In contrast to P90, the P30 treatment exhibited a 56–321% increase in AMF colonization and elevated expression of *PPDK*, *accC*, and *FabI*. The P0 treatment, meanwhile, was characterized by a 17–24-fold increase in organic acid exudation, a 35% increase in SRL, and upregulation of the genes *pckA*, *MDH*, *aceB*, and *CS*. Cultivars differed in their adaptive preferences: AM-dependent types were better suited to moderate P limitation, whereas fine-rooted cultivars were advantageous under severe P depletion. Overall, our findings reveal the regulatory networks underlying soybean P-acquisition strategies and highlight their breeding and management significance. This study provides a foundation for developing P-efficient soybean cultivars and for precision P management in sustainable agriculture.

**Supplementary Information:**

The online version contains supplementary material available at 10.1186/s12870-025-07957-x.

## Introduction

Phosphorus (P) is an essential macronutrient required for plant nucleic acid synthesis, energy metabolism, and signal transduction, and directly influences crop productivity [1]. However, inorganic phosphate (Pi) is readily immobilized by cations such as iron and aluminum, resulting in low bioavailability of applied P fertilizers. Consequently, the in-season utilization rate of P fertilizers is only approximately 30%, representing a major limitation to sustainable agricultural production [2, 3]. Thus, enhancing the inherent ability of crops to activate and utilize soil P pools is a crucial strategy for improving P use efficiency [4].

To adapt to persistent low P environments, plants have evolved three primary P acquisition strategies [5]: (1) Root architectural modification: Increasing SRL and reducing the root diameter to expand soil exploration [6, 7]; (2) Arbuscular mycorrhizal fungal (AMF) symbiosis: Leveraging extensive fungal hyphal networks to access distant P sources [8]; and (3) Root exudate release: Enhancing secretion of organic acids and phosphatases to solubilize fixed P [9]. Critically, all three strategies consume substantial carbon (C) resources derived from photosynthesis [10]. This C investment inevitably creates trade-offs in resource allocation among these strategies [11], and ignoring this balance in breeding risks overemphasizing any single trait while overlooking genotypes that maintain higher whole-plant efficiency and stable yield across diverse P environments. For example, fine-rooted cultivars tend to invest less carbon in AMF symbiosis, resulting in higher fertilizer requirements to sustain yield [12]. Understanding how plants deploy P uptake strategies is thus critical for improving crop P-use efficiency [13].

Rather than being fixed traits, plant P acquisition strategies are environmentally regulated, particularly by the degree of P limitation. Under low P stress, plants divert photosynthates to roots, increasing the root-to-shoot ratio (R/S) and triggering divergent phenotypic adaptations; for example, some species develop longer, thinner roots to enhance soil exploration, whereas coarse-rooted species invest more in maintaining AMF symbiosis [14]. By contrast, the relationship between root exudation and root architecture is more complex. Across species and environmental contexts, correlations between root exudation and specific root length (SRL) have been reported as negative, positive, or non-significant, and this complexity is likely modulated by soil P availability [15]. Arbuscular mycorrhizal colonization generally reduces organic acid exudation [16]. However, as P limitation intensifies, AM fungal hyphae themselves may fail to obtain sufficient P, and root exudation becomes the dominant strategy for P acquisition [17]. These contrasting P acquisition strategies align with ecological niche differentiation along global P gradients. AMF-dependent species are common in moderately P-limited soils (e.g., subtropical or tropical forests) [18]. Cluster-rooted species with strong root exudates dominate in ancient, severely P-depleted soils (e.g., Australia and South Africa) [19, 20].

Classical physiological and morphological assays provide a critical phenotypic foundation for distinct phosphorus acquisition strategies. Molecular studies have identified key components, such as the SPX–PHR hub for phosphate signaling [19, 21], the *RAM1/WRI1-like* module for AM symbiosis [8, 22], and enzymes such as PEPC and MDH for organic acid exudation [23]. However, a gene-by-gene perspective cannot fully capture the system-level coordination among these strategies. Integrated transcriptomic and metabolomic analyses across soil-P gradients are therefore needed to define the molecular signatures and regulatory modules underlying P-acquisition strategy shifts.

Soybean (*Glycine max* L.) is an important component of sustainable agroecosystems but is highly sensitive to P deficiency [24]. As soybean cultivation expands into low P regions such as southern China and Brazil, inadequate P availability increasingly constrains yield potential [16]. As a typical coarse-rooted species, soybean exhibits a root architecture that favors the establishment of arbuscular mycorrhizal symbiosis. Soybean can also respond to low P levels by increasing root length and reducing diameter [25]. However, the way these morphological adjustments are coordinated with AMF colonization and root exudate secretion remains unclear. Thus, clarifying how root architecture, AMF colonization, and root exudation are coordinated across the soil-P gradient in soybean remains a key knowledge gap.

To address this gap, we investigated physiological and morphological responses of five soybean cultivars grown across a soil P gradient (0–120 mg P kg⁻¹). For representative P regimes and contrasting genotypes, we further profiled root transcriptomes and metabolites. We hypothesized that (1) soil P availability is associated with the strategic shift between root-based and symbiosis-based P acquisition in soybean; and (2) genotypic differences in root architecture underlie distinct preferences, with coarse-rooted cultivars primarily relying on AMF symbiosis, and fine-rooted cultivars exhibiting greater morphological plasticity to support root-driven acquisition under severe stress.

## Materials and methods

### Experimental design

On the basis of previous studies [25], five soybean cultivars were selected: Qiandou 11 (Qd11, coarse), Aixuan (Ax, fine roots), and Zhonghuang 13 (Zh13, coarse) as P-efficient genotypes; Niumao (Nm, fine roots) as a P-sensitive genotype; and Williams 82 (Wm82, coarse) as the reference genotype. The experiment was conducted in a greenhouse at Guizhou University, Guiyang, Guizhou Province, China. Soil was collected from Qiannan Prefecture, Guizhou (26°17′N, 106°45′E) and represents typical P-deficient leached soil. The total and available P contents were 0.32 g kg⁻¹ and 3 mg kg⁻¹, respectively. Additional soil properties are listed in Additional file 1: Table S1. Plastic pots (30 cm top diameter, 20 cm height, and 20 cm base diameter) were filled with 7.5 kg of air-dried soil.

Five P levels were applied: 0 mg P kg⁻¹ (severe deficiency, P0), 30 mg P kg⁻¹ (moderate deficiency, P30), 60 mg P kg⁻¹ (mild deficiency, P60), 90 mg P kg⁻¹ (adequate, P90), and 120 mg P kg⁻¹ (excess, P120). P was supplied as KH₂PO₄, and potassium levels were equalized via KCl. A complete nutrient mixture was used to supply other elements and prevent nutrient deficiencies (Additional file 1: Table S2) [26]. A completely randomized two-factor design was adopted with three replicates per treatment, resulting in 75 pots.

### Plant growth and sample collection

The seedlings were thinned to four plants per pot at the first trifoliate leaf stage. The P treatments commenced once two true leaves had developed. The plants were irrigated every four days (500 mL per pot) for 30 days. At harvest, the roots and shoots were rinsed with distilled water and blotted dry. The samples for physiological analysis were stored at 4 °C, whereas those for transcriptomic and metabolomic analyses were flash-frozen in liquid nitrogen and stored at − 80 °C.

### Plant biomass and P contents

The samples were initially inactivated at 105 °C for 30 min, followed by drying at 75 °C to a constant weight. The dry biomass was measured using an analytical balance. The samples were ground and sieved through a 0.5 mm mesh. The P concentration was determined following H₂SO₄ and H₂O₂ digestion via the vanadium-molybdenum yellow colorimetric method [27]. P accumulation (mg plant⁻¹) was calculated as the product of biomass and P concentration.

### Root morphology

The entire root system, including both absorptive and structural roots, was scanned from each pot using an Epson PV850 Pro scanner (Epson, Long Beach, CA, USA) and analyzed using WinRHIZO 2019 Pro software (Regent Instruments, Quebec, Canada).

### Root acid phosphatase activity and organic acids exudation

Acid phosphatase (APase) activity was measured as described by Shen [28]. The roots were incubated in a solution of p-nitrophenyl phosphate (pNPP) disodium salt at 25 °C for 60 min in the dark. The reaction was terminated by adding 1 mL of 1 mol/L NaOH, and the absorbance was recorded at 405 nm.

Organic acids exudation was quantified following Liu [29]. The roots were incubated in 100 mL of 0.5 mol/L CaCl₂ solution for 4 h in the dark. The solution was filtered through a 0.22 μm membrane and stored at − 20 °C [30]. Organic acids were identified and quantified via an Agilent 1260 Infinity II HPLC system (Agilent Technologies, Santa Clara, CA, USA) equipped with a ZORBAX SB-C18 column (4.6 × 250 mm, 5 μm).

### Root mycorrhizal colonization

Approximately 1-cm long segments were cleared in 10% KOH at 90 °C, stained with 0.05% trypan blue, and decolorized in a lactoglycerol solution (lactic acid: glycerol: water = 1:1:1). Thirty stained root segments per replicate were randomly selected and mounted in 30% glycerol for microscopic observation via an Olympus BX51 microscope (Olympus, Tokyo, Japan). Mycorrhizal colonization was assessed via the gridline intersect method [31].

### Metabolomic analysis

Root metabolite extraction was performed as described by Li [32], with a quality control sample inserted every nine test samples. Metabolomic profiling was conducted via ultrahigh-performance liquid chromatography coupled with Fourier transform mass spectrometry (UHPLC-Q Exactive HF-X, Thermo Fisher Scientific). The chromatographic and MS conditions followed those of Zhou [33]. Metabolites with a relative standard deviation > 30% were excluded. Compound identification was based on matches to HMDB (http://www.hmdb.ca/), METLIN (https://metlin.scripps.edu/), and an in-house database (Majorbio). A total of 1,072 and 1,117 metabolites were identified in positive and negative ion modes, respectively (Additional file 1: Table S3). Differentially accumulated metabolites (DAMs) were defined by a VIP > 1 and *P* < 0.05.

### Transcriptomic analysis

Total RNA was extracted from roots via the MJZol Total RNA Extraction Kit (Majorbio, Shanghai, China) and purified via the RNA Purification Kit (Majorbio). The RNA purity and concentration were measured via a NanoDrop 2000. Samples with OD260/280 ≥ 1.8 and OD260/230 ≥ 1.0 were retained. RNA integrity was confirmed by agarose gel electrophoresis. Only samples with ≥ 1 µg total RNA and concentrations ≥ 35 ng µL⁻¹ were used for sequencing.

cDNA libraries were prepared via the Illumina TruSeq RNA Sample Prep Kit and sequenced on the NovaSeq X Plus platform (Illumina, Hayward, CA, USA). Each sample yielded > 6.15 Gb of clean data with a Q30 ≥ 93.46% (Additional file 1: Table S4). Clean reads were mapped to the soybean reference genome (Gmax 508 Wm82.a4.v1) via HISAT2 (v2.1.0), with mapping rates exceeding 88.7% (Additional file 1: Table S5). Transcript assembly was performed via Cufflinks (v2.2.1) [34]. Differentially expressed genes (DEGs) were identified via DESeq2 with significance criteria of adjusted *P* < 0.05 and |log₂FC| ≥ 1. Gene Ontology (GO) and KEGG pathway annotations were performed for the DEG sets. GO enrichment was conducted via GOATOOLS (v0.6.5) [35], and KEGG enrichment was conducted via gseapy [36] in conjunction with scipy [22]. Weighted gene coexpression network analysis (WGCNA) and visualization were conducted via the Majorbio Cloud Platform (https://report.majorbio.com).

### Statistical analysis

Two-way ANOVA was performed using the agricolae package in R (v4.3.0) to evaluate the effects of P level, genotype, and their interaction. When significant effects were detected, Duncan’s multiple range test (*P* < 0.05) was applied using the agricolae::duncan.test function. Pearson correlation analysis was used to examine relationships among root traits, AMF colonization, and organic acids exudation.

Principal Coordinate Analysis (PCoA) was conducted using the vegan package in R (v4.3.0), based on Bray–Curtis dissimilarity matrices. Structural Equation Modeling (SEM) was performed using SPSSPRO (https://www.spsspro.com), with the model including SRL, root diameter, organic acids exudation, AMF colonization, and P accumulation. Model fit was assessed using root mean square error of approximation (RMSEA), goodness-of-fit index GFI, and normed fit index (NFI).

Figures were generated using Origin 2021 (OriginLab, Northampton, MA, USA), and network diagrams were visualized using Cytoscape v3.9.1. All data are presented as mean ± SE. Prior to regression, outliers were identified based on pre-specified biological and diagnostic criteria and were excluded from regression fitting; the excluded data points are shown as open circles in Fig. 3.

## Results

### Effects of P levels on biomass and P accumulation

**Fig. 1:**
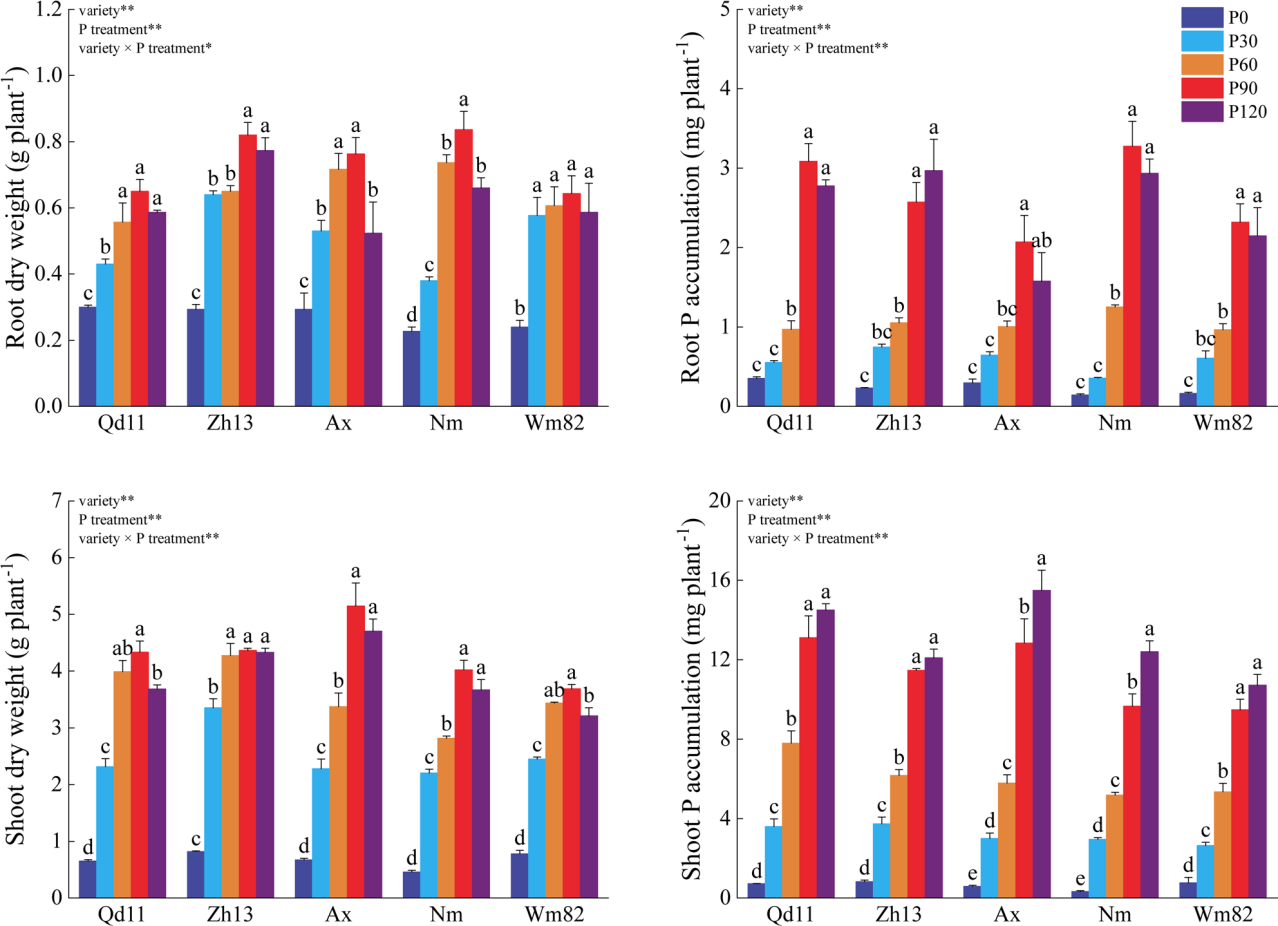
Effects of soil P availability on soybean growth and P accumulation. Soybean shoot and root biomass, as well as tissue P concentrations, were measured under different soil P levels. Bars represent mean ± SE (n = 3). Asterisks denote significant differences between treatments (*P < 0.05; **P < 0.01). Different lowercase letters indicate significant differences among cultivars within the same treatment (*P* < 0.05). Cultivar abbreviations: Qd11 (Qiandou11), Zh13 (Zhonghuang13), Ax (Aixuan), Nm (Niumao), and Wm82 (Williams 82)

As P supply increased, both shoot and root biomass of soybean plants generally rose and peaked under the P90 treatment (shoot: 3.6–5.1 g plant⁻¹; root: 0.64–0.83 g plant⁻¹). Compared to P90, root biomass under P120 decreased significantly by 45.9% in Ax and 26.7% in Nm (*P* < 0.05; Fig. 1A), while shoot biomass in Qd11 and Wm82 declined significantly by 17.5% and 14.9%, respectively (*P* < 0.05; Fig. 1C). The trend in P accumulation in root tissue was largely consistent with that of biomass: all genotypes except Zh13 reached their maximum value under P90, ranging from 2.07 to 3.28 mg plant⁻¹ (Fig. 1B). In contrast, shoot P accumulation continued to increase with rising P supply, peaking under P120 at 10.72–15.50 mg plant⁻¹ (Fig. 1D). Notably, under both P90 and P120, Ax exhibited lower root P accumulation but higher shoot P accumulation compared to other genotypes, indicating a pronounced shoot P enrichment trait under higher P levels.

**Fig. 2:**
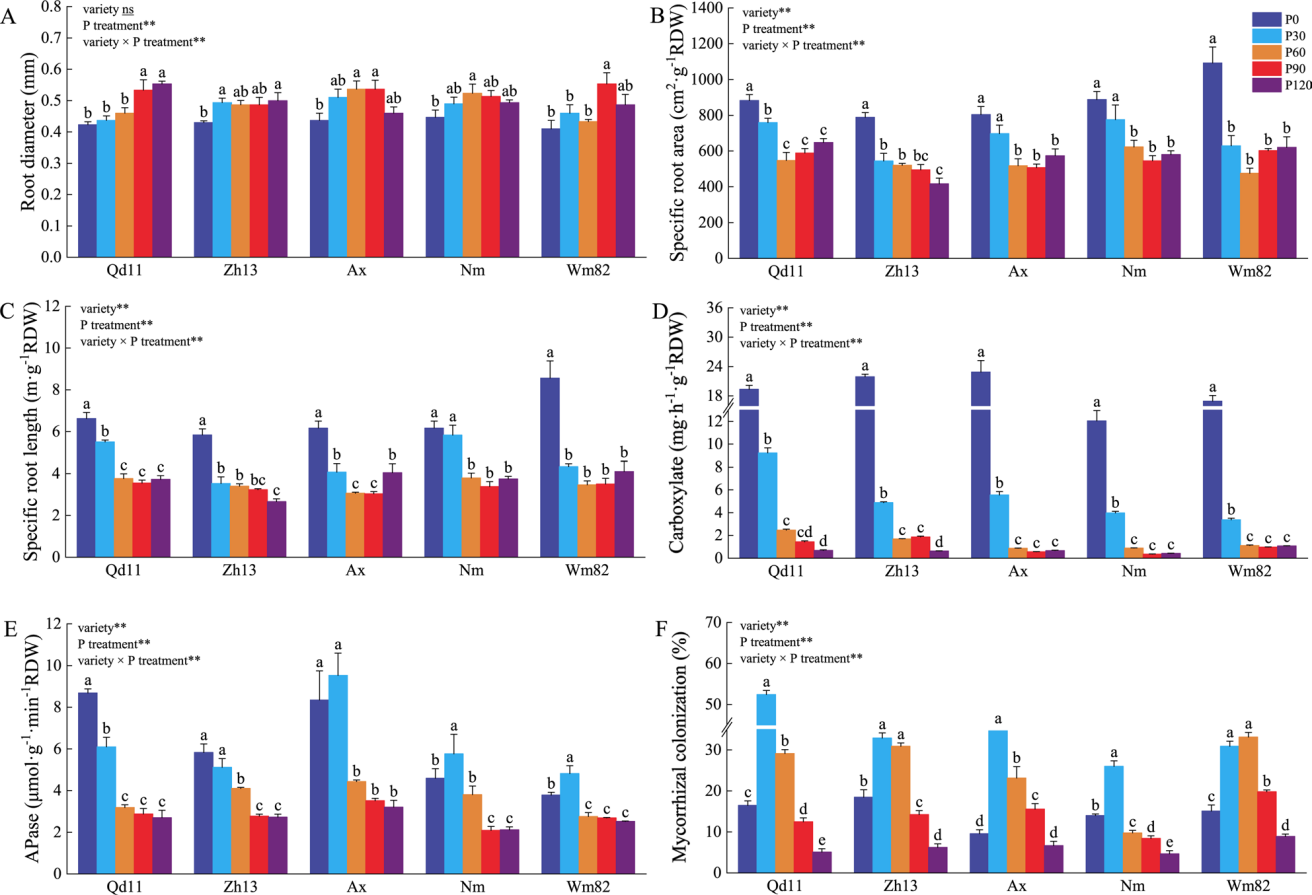
Effects of P supply on root morphology, exudates, and mycorrhizal colonization. **A** Root diameter (RD). **B** Specific root area (SRA). **C** Specific root length (SRL). **D** Organic acids exudation. **E** Acid phosphatase (APase) activity. **F** AMF colonization rate. Data are mean ± SE (n = 3). Different lowercase letters indicate significant differences among cultivars within the same P level (P < 0.05). Cultivar abbreviations are as in Fig. 1

P application significantly influenced root morphology (*P* < 0.01, Fig. 2A, Table S6). The varieties Qd11 and Wm82 exhibited higher sensitivity to P application changes: when the P application was ≤ 60 mg P kg⁻¹, their root diameter significantly decreased, whereas in the other varieties, the significant decrease in root diameter occurred under extremely low P conditions (P0). In contrast, specific root area (SRA; Fig. 2B), SRL (Fig. 2C), root-to-shoot ratio (Table S6), and APase activity (Fig. 2E) all increased with decreasing P supply and reached their highest values under P0 conditions. Consistent with the above patterns, organic acids exudation was strongly induced by P deficiency, with its secretion under P0 being 17 to 24 times higher than that under P90 (Fig. 2D; Table S7).

**Fig. 3:**
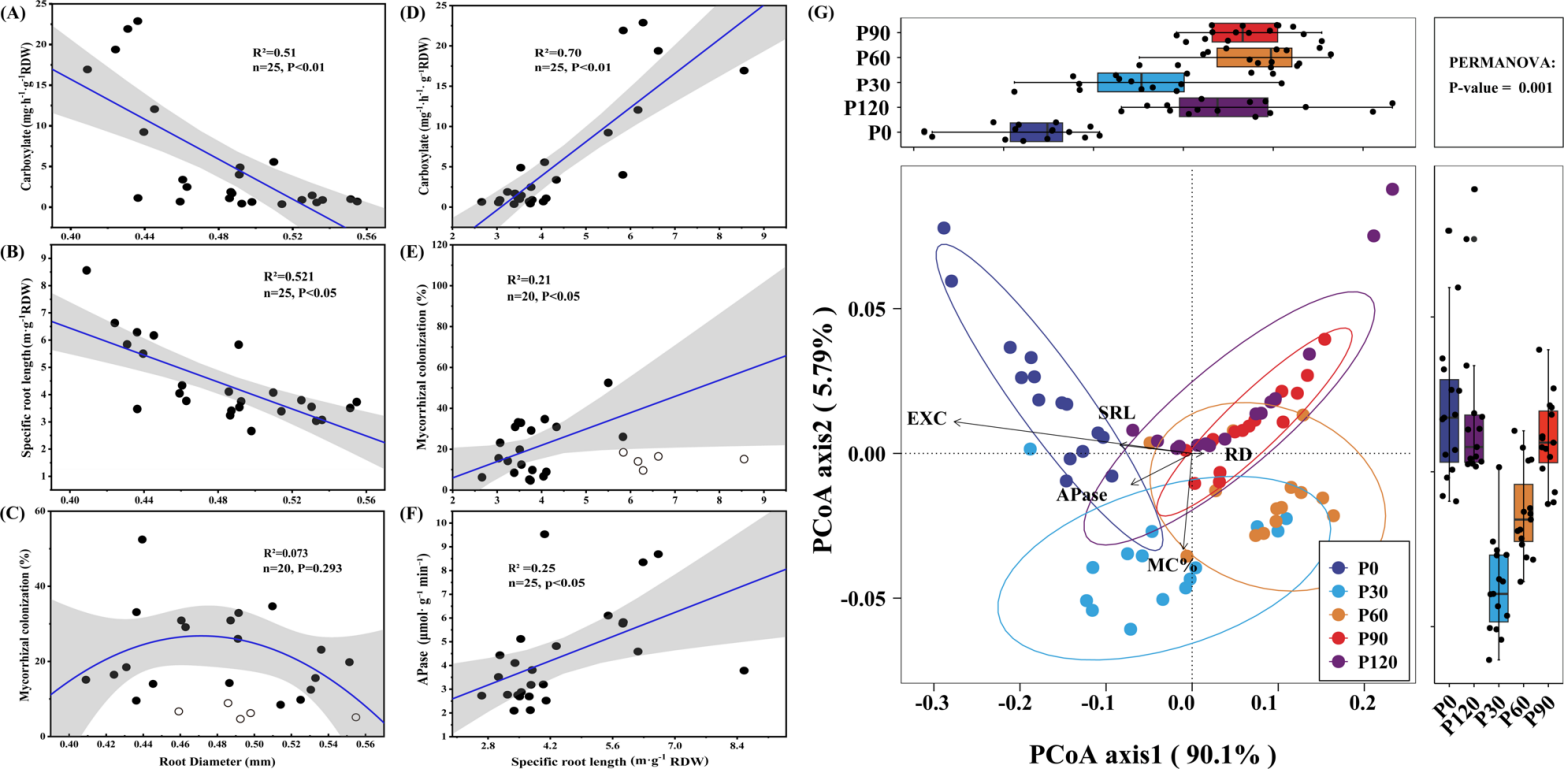
Correlation and ordination analyses of P uptake strategies. **A**-**F** Correlation analysis among uptake-related traits. **G** Principal coordinate analysis (PCoA) showing divergence of uptake strategies across P treatments. Open circles indicate data points excluded from regression fitting; specifically, points from the root diameter–AMF colonization relation at P120 and the SRL–AMF colonization relation at P0

The changes in mycorrhizal colonization rate were different from those of root morphology and exudates (Fig. 2F): within the P application range from P120 to P30, the mycorrhizal colonization rate significantly increased as P application decreased, reaching a peak at P30 (30–50%). However, when P application dropped to P0, the colonization rate significantly decreased to 10–20%. Correlation analysis indicated that root diameter was significantly negatively correlated with organic acids exudation (R² = 0.50, *P* < 0.01; Fig. 3A), while its relationship with mycorrhizal colonization was weak and not significant (R² = 0.073, *P* = 0.293; Fig. 3C). SRL was positively correlated with organic acids exudation (R² = 0.25, *P* < 0.05; Fig. 3D), AMF colonization (R² = 0.21, *P* < 0.01; Fig. 3E), and APase activity (R² = 0.25, *P* < 0.05; Fig. 3F). PCoA results (Fig. 3G) showed that PCoA1 explained 90.1% of the variation in P acquisition strategies. Organic acids exudation was the dominant factor of PCoA1, with APase activity, SRL, and root diameter also highly correlated. Mycorrhizal colonization was the dominant factor for PCoA2 (accounting for 5.7% of variation). Samples from P0 and P30 clustered on the left axis of PCoA1, while samples from other P treatments were mainly distributed on the right axis (Fig. 3G). PERMANOVA results were consistent with those of the PCoA analysis, showing that the differences between P0/P30 and other P levels were highly significant (*P* < 0.01, Table 1), indicating that the P availability gradient was associated with a shift in soybean P acquisition strategies.

**Table 1:** Effects of P supply on soybean P acquisition strategies based on PERMANOVA

Group	P0/P30	P0/P60	P0/P90	P0/P120	P30/P60	P30/P90	P30/P120	P60/P90	P60/P120	P90/P120
Variation	0.47	0.78	0.80	0.67	0.37	0.42	0.31	0.05	0.11	0.04
P value	0.001	0.001	0.001	0.001	0.001	0.001	0.003	0.226	0.058	0.282

**Fig. 4:**
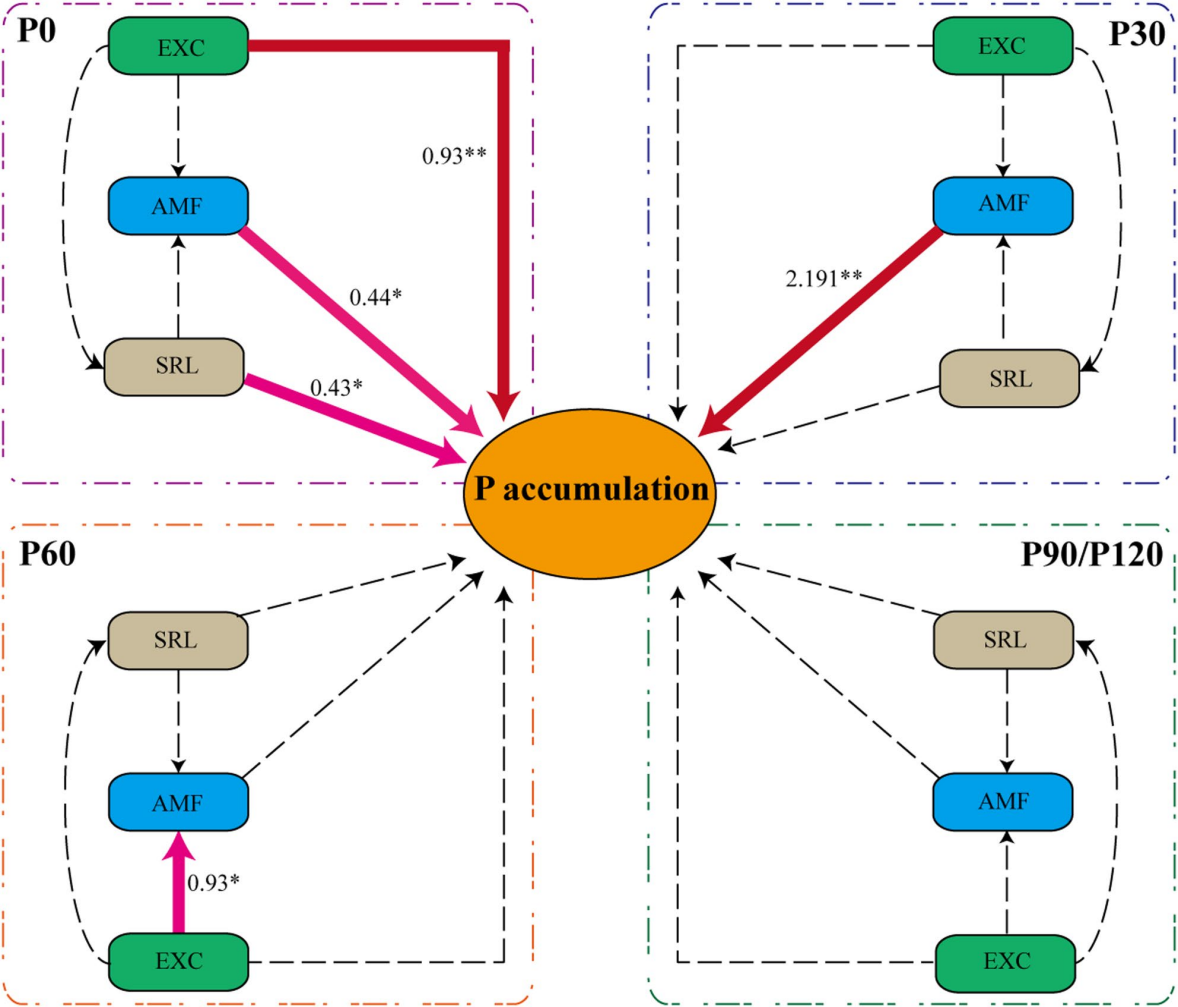
Structural equation model (SEM) of P uptake strategies in soybean roots. Pink arrows indicate significant positive paths (*P* < 0.05), red arrows indicate highly significant positive paths (*P* < 0.01), and dotted lines indicate non-significant paths (*P* > 0.05). Asterisks denote significance: *P < 0.05; **P < 0.01. Model fit: RMSEA < 0.001; GFI = 0.902; NFI = 0.902

Under P0 conditions, soybean primarily relies on a root-based activation strategy, as indicated by the significant increase in root exudates and morphological plasticity (e.g., high SRL) (Fig. 3B). Pathway analysis showed that the secretion of organic acids made the greatest positive contribution to P accumulation (path coefficient = 0.93), which was stronger than that of AMF (Fig. 4). Under P30 (30 mg P kg⁻¹, moderate low P) conditions, soybean shifted to rely more on the symbiotic strategy (Fig. 4), showing the highest mycorrhizal colonization rate (Fig. 2F), with only the mycorrhizal colonization rate being significantly correlated with P accumulation (path coefficient = 2.191; Fig. 4). Under P60-P120 (≥ 60 mg P kg⁻¹) conditions, the dependence on root exudates and mycorrhizal symbiosis gradually decreased, and root P acquisition became more reliant on direct ion absorption.

### Metabolomic signatures of P-dependent strategy shifts

To elucidate the metabolic mechanisms underlying P acquisition strategy shifts, particularly the root-autonomous activation observed under P0 and the symbiosis-dependent strategy under P30, we profiled root metabolomes at three P levels: P0 (representing rhizosphere activation and morphological plasticity), P30 (AM symbiosis), and P90 (adequate-P control). Three soybean genotypes were analyzed: Ax (P-efficient), Nm (P-inefficient), and Wm82 (reference).

**Fig. 5:**
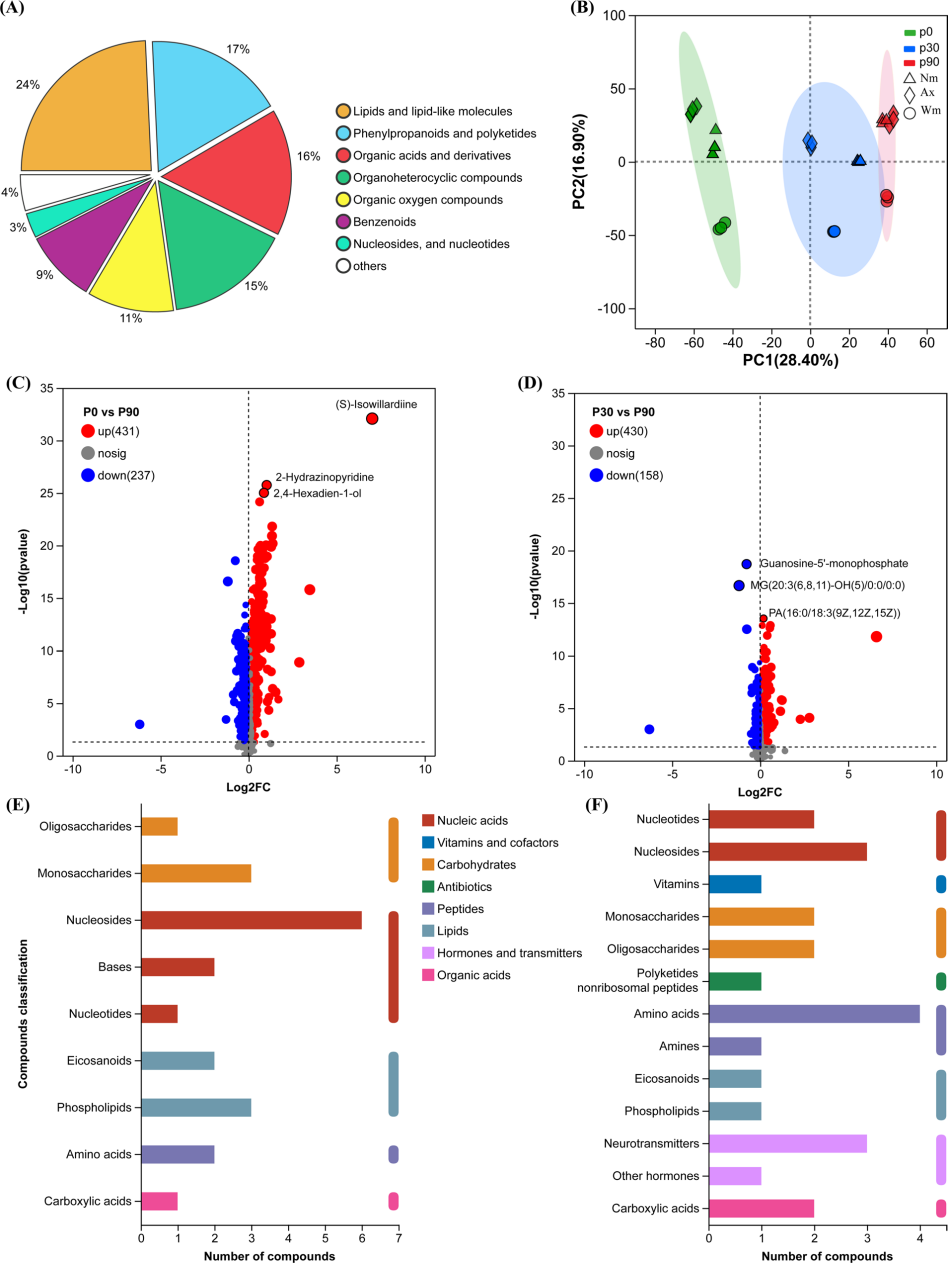
P-induced changes in soybean root metabolites. **A** Classification of differential metabolites based on the Human Metabolome Database (HMDB). Category proportions in panel A were computed from a pooled set across P treatments after aggregating Ax, Nm, and Wm82. **B** Principal component analysis (PCA) of metabolite profiles. Cultivar identity is indicated on each point. **C**–**D** Volcano plots comparing P30 vs. P90 (**C**) and P0 vs. P90 (**D**). **E**–**F** Classification statistics of differential metabolites in P0 vs. P30 (**E**) and P30 vs. P90 (**F**)

Soybean root differentially accumulated metabolites were classified using the HMDB. Ranked by proportion, the dominant classes were lipids and lipid-like molecules (24%), phenylpropanoids and polyketides (17%), organic acids and derivatives (16%), organoheterocyclic compounds (15%), and organic oxygen compounds (11%) (Fig. 5A). Principal component analysis (PCA) resolved treatment structure along the P gradient (Fig. 5B): PC1 explained 28.4% of the variance (P0 on the negative axis, P90 on the positive), and the first two PCs explained 45.3% in total, with P30 in between.

Under P0, 431 differentially accumulated metabolites (DAMs) were identified, including organic acids (e.g., malate, citrate) and lipid species (e.g., phospholipids and long-chain acyl derivatives or eicosanoid-like compounds). The accumulation of these metabolites is consistent with enhanced organic acid exudation and membrane remodeling under severe limitation.

Under P30, 588 DAMs were identified (430 upregulated and 158 downregulated, Fig. 5C). Compared with P0, P30 showed more pronounced changes in lipid species, especially fatty acids and glycerophospholipids, which may provide carbon to support AM symbiosis. Consistently, DAMs at P30 were enriched in pathways including ABC transporters, flavonoid biosynthesis, and glycerophospholipid metabolism, collectively supporting a symbiosis-dependent strategy.

KEGG pathway enrichment analysis was consistent with changes in metabolic pathways related to P acquisition. At P0, the tricarboxylic acid (TCA) cycle was significantly enriched, consistent with the higher accumulation of organic acids. At P30, linoleic acid metabolism was markedly enriched (Additional file 2: Fig. S2), consistent with increased capacity for fatty acid metabolism that may support AMF symbiosis. Notably, flavonoid biosynthesis was enriched at both P0 and P30.

### Transcriptomic responses to varying soil P availability

The distinct metabolic profiles between P0 and P30 suggested fundamentally different carbon flux patterns. To elucidate the transcriptional basis underlying these differences, we performed transcriptomic analyses under the same treatments.

DESeq2 analysis revealed a greater number of differentially expressed genes (DEGs) under P0 than under P30 across all genotypes (Additional file 2: Fig. S3A). Wm82 presented the greatest transcriptomic response under P0, with 11,506 DEGs (4,351 upregulated and 7,155 downregulated). PCA revealed that the P0-treated samples clustered along the negative axis of PC1, whereas the P90 samples were on the positive axis. The P30 samples were scattered in between (Additional file 2: Fig. S3B). These results were corroborated by PERMANOVA (*P* < 0.01), indicating that increasing P deficiency is associated with a broader transcriptional response. Notably, we identified a gene, *GmPHT1-11*, associated with arbuscular mycorrhizal (AM) symbiosis, which exhibited a strong transcriptional response to P availability. Compared to P90, the expression of Nm, Ax, and Wm82 was upregulated by 4.5, 8.0, and 11.2-fold (*P* < 0.01) in P30, respectively (Additional file 2: Table S8).

KEGG enrichment analysis revealed the top 30 significantly enriched pathways (Additional file 2: Fig. S3C). In the P0 vs. P90 comparison, all genotypes showed enrichment in pathways such as starch and sucrose metabolism, glycolysis/gluconeogenesis, and alanine, aspartate, and glutamate metabolism. In contrast, P30 vs. P90 comparisons were enriched in carotenoid biosynthesis, glycerophospholipid metabolism, ABC transporters, and plant hormone signal transduction. Compared with the P-inefficient Nm, the P-efficient genotypes (Ax and Wm82) showed greater enrichment in isoflavonoid biosynthesis, plant-pathogen interaction, and mitogen-activated protein kinase signaling pathways, findings that may explain their higher AMF colonization rates (Fig. 2F).

**Fig. 6:**
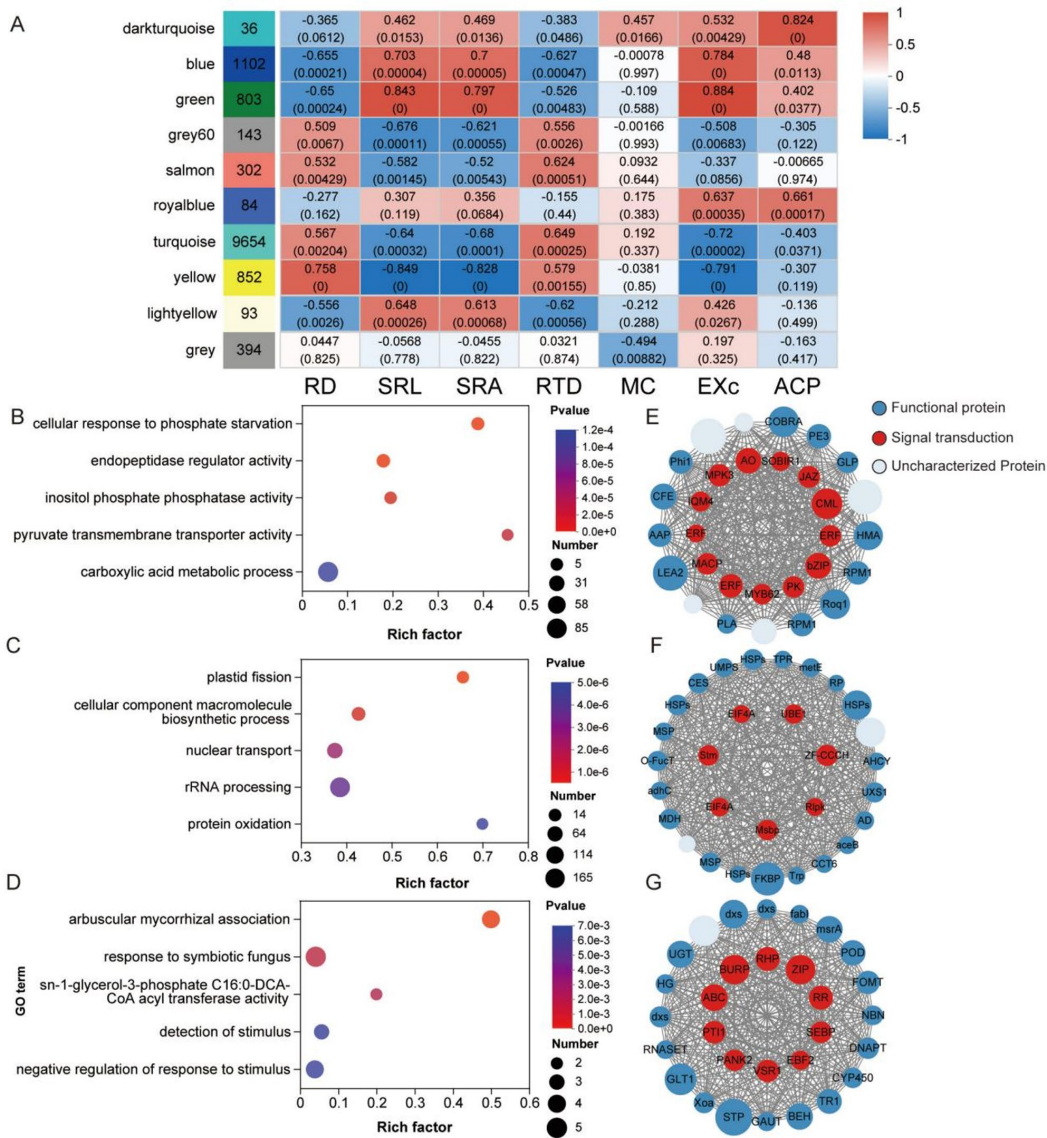
Weighted gene co-expression network analysis (WGCNA) of root DEGs. **A** Correlations between gene co-expression modules and root traits. Abbreviations: RD, root diameter; SRL, specific root length; SRA, specific root area; RTD, root tissue density; MC, mycorrhizal colonization; EXC, organicacid exudation; ACP, acid phosphatase activity. Only modules significantly correlated with uptake traits are shown. **B**–**G** GO enrichment and network analyses of representative modules (blue, green, light yellow, turquoise, yellow, grey60, salmon, dark turquoise, and grey)

To identify regulatory modules and hub genes associated with P acquisition strategies, we performed weighted gene co-expression network analysis (WGCNA). Modules positively correlated with SRL and root exudation (e.g., blue, green and turquoise) were enriched in carboxylic acid metabolism. Genes such as *pckA* and *MDH* in these modules showed higher expression under P0. In contrast, AM-associated modules (e.g., dark turquoise and grey) showed higher expression of fatty-acid biosynthesis genes, including *accA/B/C*,* FabF*,* FabI* and *FATB* under P30 (Fig. 6G), which is consistent with increased investment in lipid metabolism that may support AM symbiosis.

### Integrated transcriptomic and metabolomic analysis

**Fig. 7:**
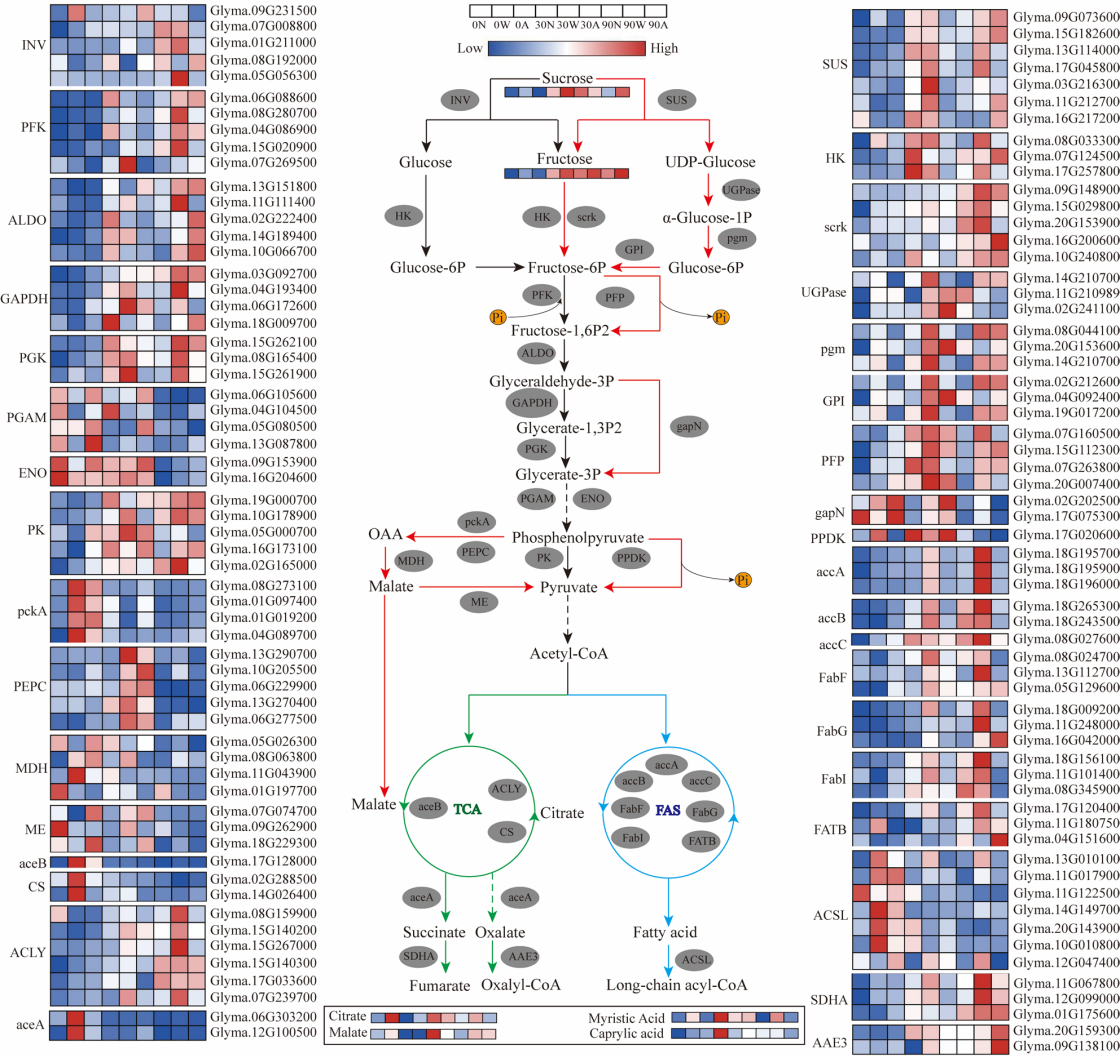
Carbon metabolism pathways in soybean roots under different P levels. Key enzymes and pathways involved in sucrose degradation, glycolysis, the tricarboxylic acid cycle, and fatty acid metabolism are highlighted. Abbreviations: INV, invertase; HK, hexokinase; SUS, sucrose synthase; Scrk, fructokinase; UGPase, UDP-glucose pyrophosphorylase; PGM, phosphoglucomutase; GPI, glucose-6-phosphate isomerase; PFK, phosphofructokinase; PFP, diphosphate-dependent phosphofructokinase; ALDO, fructose-bisphosphate aldolase; GAPDH, glyceraldehyde-3-phosphate dehydrogenase; PGK, phosphoglycerate kinase; GapN, non-phosphorylating GAPDH; PGAM, phosphoglycerate mutase; ENO, enolase; PK, pyruvate kinase; PPDK, pyruvate phosphate dikinase; PckA, phosphoenolpyruvate carboxykinase; PEPC, phosphoenolpyruvate carboxylase; OAA, oxaloacetate; MDH, malate dehydrogenase; ME, malic enzyme; AceB, malate synthase; CS, citrate synthase; ACLY, ATP citrate lyase; AceA, isocitrate lyase; SDHA, succinate dehydrogenase; AAE3, oxalyl-CoA ligase; accA/B/C, acetyl-CoA carboxylase subunits; FabF/I/G, fatty-acid biosynthesis enzymes; FATB, acyl-ACP thioesterase; ACSL, acyl-CoA synthetase long-chain family. Black arrows indicate standard pathways; red, alternative pathways; green, TCA cycle; blue, fatty acid cycle; solid lines, direct pathways; dotted lines, indirect pathways

Analysis of the glycolytic pathway (Fig. 7) showed patterns consistent with a bypass involving PEPC: phosphoenolpyruvate (PEP) may be converted to oxaloacetate and malate via PEPC, *pckA* and *MDH*, with the corresponding genes showing an expression gradient of P0 > P30 > P90. In contrast, the PK branch converts PEP to pyruvate, supplying acetyl-CoA/malonyl-CoA for fatty-acid biosynthesis (*accA/B/C*,* FabF/I*,* FATB*). Under Pi stress, PPi-dependent bypass enzymes such as PFP and PPDK may help conserve ATP/Pi and may contribute to maintaining glycolytic flux. Genes such as *accA/B* and *FabI* showed a reversed trend (P30 > P90 > P0). As shown in Fig. 7, soil P availability is associated with differential use at the pyruvate branch, with patterns consistent with greater engagement of pathways related to carboxylic acid metabolism under severe deficiency and increased fatty acid biosynthetic capacity that may support symbiosis under moderate deficiency.

## Discussion

The P acquisition strategy of crops is a key intrinsic mechanism for their adaptation to soil environments and for enhancing nutrient use efficiency. By integrating physiological phenotyping with root metabolomic and transcriptomic data, this study demonstrates that across a soil P gradient, soybean does not rely on a single, fixed P acquisition mode. Instead, as P availability decreases, the dominant strategy shifts in a regime-like manner. At high P supply (P120), P acquisition is primarily driven by direct uptake via root surface transporters. At moderate P levels (P60–P30), soybean increasingly relies on AMF symbiosis. Under severe P deficiency (P0), P acquisition is dominated by a root-foraging strategy characterized by increased SRL and enhanced root exudation (Figs. 3G and 4). The gradient in soil P concentration acts as a major environmental driver of this transition. Furthermore, the plant’s inherent genetic traits, particularly root system architecture, profoundly influence strategic preferences. Among the genotypes studied, those with coarser root systems (Wm82, Qd11, and Zh13) exhibited greater dependence on AMF symbiosis across a wider P range (Fig. 2F), whereas the fine-rooted genotype (Ax) demonstrated stronger root-foraging capacity under severe P deficiency (Fig. 2D). Therefore, the ultimate P acquisition performance in soybean can be understood as the outcome of interactions between external soil P levels and internal genotypic differences, primarily represented by root system architecture.

Under moderate P deficiency (P60–P30), soybean shifted its P acquisition pattern away from sole dependence on direct root uptake toward a greater reliance on the “outsourcing” strategy of arbuscular mycorrhizal symbiosis. As soil P supply decreased from P90 to P30, shoot and root P concentrations in all genotypes declined significantly (Fig. 1B, D; *P* < 0.05), indicating a transition from P sufficiency to P stress and the activation of adaptive responses. Within the P120–P30 range, AMF colonization increased significantly with decreasing P availability, peaking at P30 (Fig. 2F; *P* < 0.05). In contrast to the rapid response of AMF symbiosis, which was already significant at P60, changes in SRL and organic acid exudation were more delayed: at P60, neither SRL nor organic acid exudation differed significantly from P90 levels in any genotype (Fig. 2B–D; *P* > 0.05). Even at P30, although organic acid secretion had increased across all genotypes (Fig. 2D; *P* < 0.05), only Qd11, Ax, and Nm showed significantly higher SRL than at P90 (Fig. 2B; *P* < 0.05). PERMANOVA and PCoA further confirmed that P30 samples were clearly separated from P120, P90, and P0 (Table 1; *P* < 0.05), supporting the characterization of moderate P deficiency as a phase dominated by enhanced AMF symbiosis, prior to the full activation of root-foraging traits.

PCA revealed that the metabolic profile of P30 plants was intermediate between P90 and P0, yet distinct from P90, with over 588 differentially accumulated metabolites significantly enriched in pathways related to lipid-associated processes (Fig. 5E, Additional file 2: Fig S2). Transcriptomic analysis further showed that key genes involved in fatty acid biosynthesis (*accA/B/C*, *FabF*,* FabI*, *FATB*) and AMF symbiosis genes (e.g., *GmPHT1-11*) were significantly upregulated under P30 compared to P90 (Figs. 6A and D and 7; Table S8). Given that AM fungi are fatty acid auxotrophs and depend on host-derived lipids for carbon [37, 38], the observed lipid profiles at P30 are likely linked to mycorrhizal symbiosis. Structural equation modeling statistically supported this outsourcing strategy: at P30, the path coefficient from AMF colonization to plant P accumulation was the strongest (path coefficient = 2.191) and highly significant (*P* < 0.01), whereas the direct contributions of root morphology and organic acid exudation were relatively weak (*P* > 0.05; Fig. 4). Compared to fine-rooted crops like maize and wheat, soybean possesses a coarser root system and is considered a typical “outsourcing” [30] or “resource-conservative” species [13, 14]. Thus, expanding the P acquisition zone via mycorrhizal networks, rather than through extensive root plasticity, represents an important adaptive pathway for soybean under moderate P deficiency.

Under severe P deficiency (P0), the arbuscular mycorrhizal (AM) symbiosis strategy becomes severely constrained. Compared to the P30 treatment, AMF colonization rates declined by 50–80% across all soybean genotypes at P0 (Fig. 2F). This observation aligns with findings in other species such as maize [37, 38] and wheat [39]. This limitation may be attributed to two factors: on one hand, AM fungal activity itself becomes constrained under extreme P deficiency; on the other hand, the carbon cost of P acquisition via mycorrhizal pathways increases substantially [10, 40]. Coupled with the fact that extreme P deficiency itself limits root carbohydrate supply (Fig. 7), this heightened cost likely makes it unsustainable for soybean to maintain the symbiosis.

In response, soybeans adopted an alternative P acquisition pathway. While fungal colonization diminished, root traits associated with a “mining strategy”, such as exudation of low-molecular-weight organic acids (Fig. 2D), SRL (Fig. 2C), and SRA (Fig. 2B), peaked under P0 conditions. Notably, organic acid concentrations in the rhizosphere were 17– to 24–fold higher at P0 than at P90 (Fig. 2D). SEM indicated that organic acid exudation had the strongest positive path to P accumulation at P0 (path coefficient = 0.93, *P* < 0.01; Fig. 4), supporting its prominence under severe deficiency (see also trait patterns in Fig. 3). Transcriptomic analysis further showed increased expression of genes involved in carboxylicacid metabolism (e.g., *pckA*, *MDH*, *ME*; Fig. 7), consistent with the higher levels of malate and other organic acids (Table S7; Fig. 5F). These compounds were detected in root exudates, which can mobilize sparingly soluble P (Fig. 2D). In parallel, energy-conserving glycolytic bypasses, including diphosphate-dependent phosphofructokinase (PFP) and PPDK, showed increased expression. This may contribute to ATP economy and internal phosphate recycling under energy-limited conditions [23, 41]. At this stage, directly enhancing root morphological plasticity, such as increased SRL and SRA, and promoting organic acids biosynthesis may become a more efficient strategy [42].

Notably, soybean genotypes exhibit considerable variation in their P sensitivity thresholds and in the trade-offs between strategies. Genotypes such as Wm82, Qd11, and Zh13 are more dependent on AMF symbiosis and maintain relatively high AMF colonization even under P-sufficient conditions (Fig. 2F), which could support higher P use efficiency and help limit fertilizer accumulation in high-total/low-available-P soils. In contrast, genotype Ax shows greater organic acid and acid-phosphatase release under P deficiency (Fig. 2D, E), suggesting suitability for soils with low total P and strong P fixation, such as acidic or alkaline soils [5]. Root system architecture plays a critical role in shaping genotype-specific P strategies (Fig. 3A). A strong positive correlation was observed between SRL and the exudation of organic acids (R^2^ = 0.70, *P* < 0.01; Fig. 3D), suggesting that fine-rooted genotypes preferentially adopt a mining strategy [30]. It should also be noted that our architectural indices were derived from whole-root scans and therefore include both absorptive and more lignified segments; consequently, they should be interpreted as integrative descriptors of root system architecture, and future work that resolves traits by root order would further refine these patterns.

These differences in P acquisition strategies reflect the underlying genetic variation in P sensing and plant–fungus signaling pathways across soybean genotypes. Considering the wide global variation in available soil P, ranging from 0.01 to 99.2 mg kg⁻¹ [43], matching soybean genotypes with soil P profiles becomes essential. Future research should focus on developing a comprehensive genotype–P–management decision framework. This dual optimization can be achieved by combining targeted breeding, such as genome editing of genes involved in pyruvate metabolic branching, with demand-based P fertilizer management.

It should be noted that the insights from this study stem from experiments conducted in a single soil type, which constrains the extrapolation of the conclusions to broader environments. To translate these findings into practical applications, future efforts should focus on establishing a genotype–soil P matching database spanning diverse agroecological zones. Such a resource would support the development of actionable, low-carbon P management strategies tailored to specific crop demands and environmental conditions. Our findings provide a foundation for precision P management and genotype–soil matching in sustainable soybean production systems. Future research should build on these correlative findings by combining ¹³C-based flux analysis with reverse genetics to directly quantify C partitioning and establish whether key candidate genes functionally drive the shifts in P-acquisition strategies.

## Conclusion

This study suggests that soil P availability is associated with strategic shifts in P acquisition in soybean. Soybean showed a higher AM symbiosis rate under moderate P deficiency. This symbiosis-based outsourcing strategy was more evident in coarse-root genotypes, which tend to exhibit lower root morphological plasticity and can benefit from mycorrhizal hyphal networks to access spatially dispersed P. Under severe P deficiency, the patterns were consistent with mining strategy, with higher expression of genes such as *pckA* and *MDH*, elevated organic acids levels, reduced root diameter, and increased SRL and SRA, which are traits associated with expanded soil exploration. Such a strategy may be particularly beneficial in P-fixing acidic soils and among fine-root genotypes.

## Supplementary Information

Supplementary Material 1. Table S1. Soil physicochemical properties. Soil organic matter (SOM), total nitrogen (TN), alkali-soluble nitrogen (AN), total P (TP), available P (AP), total potassium (TK), and available potassium (AK) measured under experimental conditions (mean ± SE, n = 3). Table S2. Nutrient solution composition. Concentrations of macronutrients and micronutrients used in the hydroponic solution for soybean cultivation. Table S3. Metabolome preconditioning results. Numbers of detected peaks, identified metabolites, and annotations in KEGG and HMDB under positive and negative ion modes. Table S4. Quality control of transcriptome sequencing. Raw and clean reads, error rate, Q20, Q30, and GC content across all samples. Table S5. Summary of transcriptome sequence alignment. Mapping statistics including total mapped, uniquely mapped, and multiply mapped reads for each sample. Table S6. Effects of P supply on root morphology. Average root diameter, root tissue density, and root-to-shoot ratio of soybean cultivars under five P treatments (P0–P120). Different lowercase letters denote significant differences at *P* < 0.05. Table S7. Root exudation of organic acids under P treatments. Secretion rates of oxalic, acetic, citric, succinic, malic, and tartaric acids across cultivars. Table S8. Expression of P starvation-responsive genes. Differential expression (Padj values, FC) of PHT, PHO, PAP, PHR, and ALMT family genes in three soybean cultivars under P30 vs P90.

Supplementary Material 2. Figure S1. Effects of P availability on soybean growth. Plant height and stem diameter under different P treatments. Lowercase letters indicate significant differences among treatments within the same cultivar (*P* < 0.05). Asterisks denote significance at *P < 0.05 and **P < 0.01. Figure S2. KEGG enrichment of differential metabolites. KEGG pathway enrichment of differentially accumulated metabolites (DAMs) in soybean roots under P treatments. Figure S3. KEGG enrichment of differentially expressed genes. (A) Expression profiles of root DEGs under different P treatments. (B) PCA of gene expression in three soybean cultivars. (C) KEGG pathway enrichment of DEGs in soybean roots.

## Data Availability

The raw transcriptomic sequencing data generated in this study have been deposited in the NCBI Sequence Read Archive under BioProject accession number PRJNA1129285 ( https://www.ncbi.nlm.nih.gov/bioproject/PRJNA1129285). The metabolomic datasets and other materials are available from the corresponding author upon reasonable request.

## Abbreviations

AMF: Arbuscular mycorrhizal fungi
C: Carbon
DAMs: Differentially accumulated metabolites
DEGs: Differentially expressed genes
GFI: Goodness-of-fit index
GO: Gene Ontology
HMDB: Human Metabolome Database
KEGG: Kyoto Encyclopedia of Genes and Genomes
MDH: Malate dehydrogenase
NCBI: National Center for Biotechnology Information
NFI: Normed fit index
P: Phosphorus
PCA: Principal component analysis
PCoA: Principal coordinate analysis
PEPC: Phosphoenolpyruvate carboxylase
Pi: Inorganic phosphate
PPDK: Pyruvate phosphate dikinase
PERMANOVA: Permutational Multivariate Analysis of Variance
RMSEA: Root mean square error of approximation
R/S: Root-to-shoot ratio
SRA: Specific root area
SE: Standard error
SRL: Specific root length
WGCNA: Weighted gene co-expression network analysis
SEM: Structural Equation Modeling
