# Supplementary material for: Phosphorus-dependent shifts in acquisition strategies revealed by integrated transcriptomics and metabolomics in soybean roots
Source: BMC Plant Biol. 2025 Dec 17;25:1753. doi: 10.1186/s12870-025-07957-x (PMC12751778; doi:10.1186/s12870-025-07957-x)
Supplement: Supplementary file 1 — Supplementary Material 1. Table S1. Soil physicochemical properties. Soil organic matter (SOM), total nitrogen (TN), alkali-soluble nitrogen (AN), total P (TP), available P (AP), total potassium (TK), and available potassium (AK) measured under experimental conditions (mean ± SE, n = 3). Table S2. Nutrient solution composition. Concentrations of macronutrients and micronutrients used in the hydroponic solution for soybean cultivation. Table S3. Metabolome preconditioning results. Numbers of detected peaks, identified metabolites, and annotations in KEGG and HMDB under positive and negative ion modes. Table S4. Quality control of transcriptome sequencing. Raw and clean reads, error rate, Q20, Q30, and GC content across all samples. Table S5. Summary of transcriptome sequence alignment. Mapping statistics including total mapped, uniquely mapped, and multiply mapped reads for each sample. Table S6. Effects of P supply on root morphology. Average root diameter, root tissue density, and root-to-shoot ratio of soybean cultivars under five P treatments (P0–P120). Different lowercase letters denote significant differences at P < 0.05. Table S7. Root exudation of organic acids under P treatments. Secretion rates of oxalic, acetic, citric, succinic, malic, and tartaric acids across cultivars. Table S8. Expression of P starvation-responsive genes. Differential expression (Padj values, FC) of PHT, PHO, PAP, PHR, and ALMT family genes in three soybean cultivars under P30 vs P90. [file 12870_2025_7957_MOESM1_ESM.docx]

**Table S1 Soil physico-chemical properties**

| Cultivation Systems | Content | Cultivation Systems | Content |
| --- | --- | --- | --- |
| SOM(g·kg^-1^) | 3.38 | pH | 4.95 |
| TN(g·kg^-1^) | 0.52 | AN (mg·kg^-1^) | 22 |
| TP(g·kg^-1^) | 0.32 | AP (mg·kg^-1^) | 3 |
| TK(g·kg^-1^) | 3.51 | AK (mg·kg^-1^) | 23 |

Note: SOM, soil organic matter; TN, total nitrogen; TK, total potassium; TP, total phosphorus; AN, alkaline hydrolysable nitrogen; AK, available potassium; AP, available phosphorus.

**Table S2 Nutrient solution formulas**

| Compound | Content  (mmol·L^-1)^ | Compound | Content  (mmol·L^-1^) | Compound | Content  (mmol·L^-1^) |
| --- | --- | --- | --- | --- | --- |
| KNO_3_ | 1.5 | MgCl_2_ | 0.025 | (NH_4_)_2_MoO_4_ | 0.00015 |
| Ca(NO_3_)_2_ | 1.2 | MnSO_4_ | 0.0015 | Na_2_B_4_O_7_·10H₂O | 0.0025 |
| NH_4_NO_3_ | 0.4 | (NH_4_)_2_SO_4_ | 0.3 | CoCl_2_ | 0.0001 |
| K_2_SO_4_ | 0.3 | ZnSO_4_ | 0.0015 | Fe-EDTA | 0.04 |
| MgSO_4_ | 0.5 | CuSO_4_ | 0.0005 |  |  |

**Table S3 Metabolomics feature detection and annotation summary**

| Ion mode | Total features | Identified metabolites | Library matches | KEGG mapped |
| --- | --- | --- | --- | --- |
| pos | 5521 | 1072 | 1000 | 576 |
| neg | 6139 | 1117 | 1074 | 575 |

Note: LC–MS was performed on a UHPLC–Q Exactive HF-X with an HSS T3 column under positive/negative ESI modes. Raw files were processed in Progenesis QI to generate a feature matrix (RT, m/z, peak area); metabolites were annotated by MS/MS matching to HMDB/METLIN (mass error < 10 ppm). QC pooled samples were injected every 5–15 runs to assess stability. Thresholds and statistics follow the Materials and Methods.

**Table S4 Quality control of transcriptome sequencing data**

| Sample | Raw reads | Clean reads | Error rate (%) | Q20 (%) | Q30 (%) | GC content (%) |
| --- | --- | --- | --- | --- | --- | --- |
| Nm_0_1 | 49237796 | 48683278 | 0.0248 | 98.09 | 94.47 | 44.54 |
| Nm_0_2 | 50130018 | 48603926 | 0.0255 | 97.30 | 94.87 | 44.53 |
| Nm_0_3 | 43197030 | 42700086 | 0.0247 | 98.09 | 94.50 | 44.18 |
| Wm_0_1 | 42793010 | 42331688 | 0.0249 | 98.05 | 94.34 | 44.75 |
| Wm_0_2 | 42725534 | 42285872 | 0.0249 | 98.03 | 94.32 | 44.68 |
| Wm_0_3 | 42776324 | 42351066 | 0.0252 | 97.91 | 93.99 | 44.68 |
| Wm_30_1 | 45733736 | 45238624 | 0.025 | 97.99 | 94.24 | 45.37 |
| Wm_30_2 | 43087380 | 42741628 | 0.0248 | 98.09 | 94.43 | 45.27 |
| Wm_30_3 | 42741668 | 42348118 | 0.0249 | 98.04 | 94.34 | 45.34 |
| Wm_90_1 | 43616990 | 43187514 | 0.025 | 97.98 | 94.16 | 44.75 |
| Wm_90_2 | 41531462 | 41133398 | 0.0251 | 97.95 | 94.09 | 44.71 |
| Wm_90_3 | 49344944 | 48850100 | 0.025 | 97.98 | 94.19 | 45.22 |
| Nm_30_1 | 43189686 | 42745584 | 0.0258 | 97.70 | 93.46 | 44.79 |
| Nm_30_2 | 51649792 | 51098366 | 0.0251 | 97.95 | 94.12 | 45.28 |
| Nm_30_3 | 52324408 | 51778412 | 0.0248 | 98.07 | 94.46 | 45.07 |
| Nm_90_1 | 46073612 | 45664770 | 0.025 | 97.98 | 94.17 | 44.84 |
| Nm_90_2 | 48708748 | 48279664 | 0.0252 | 97.93 | 94.02 | 44.9 |
| Nm_90_3 | 50344562 | 49861600 | 0.0251 | 97.97 | 94.15 | 44.7 |
| Ax_0_1 | 45551916 | 45097412 | 0.0249 | 98.02 | 94.29 | 44.72 |
| Ax_0_2 | 49401172 | 48730876 | 0.0253 | 97.85 | 93.91 | 44.84 |
| Ax_0_3 | 42806436 | 42366030 | 0.0256 | 97.81 | 93.66 | 44.79 |
| Ax_30_1 | 47959632 | 47524262 | 0.0247 | 98.09 | 94.49 | 44.86 |
| Ax_30_2 | 45280614 | 44803974 | 0.0247 | 98.08 | 94.50 | 45.06 |
| Ax_30_3 | 49356436 | 48893472 | 0.0252 | 97.93 | 94.03 | 45.15 |
| Ax_90_1 | 42470818 | 41991328 | 0.0251 | 97.97 | 94.19 | 45.13 |
| Ax_90_2 | 57484824 | 56933816 | 0.0249 | 98.03 | 94.32 | 45.27 |
| Ax_90_3 | 44678458 | 44259842 | 0.0247 | 98.10 | 94.50 | 45.18 |

Note: (1) Sample: Sample name; (2) Raw reads: The total number of entries in the original sequencing data; (3) Clean reads: The total number of entries in the sequencing data after quality control; (4) Error rate (%): The average error rate of sequencing bases corresponding to quality control data, generally below 0.1%; (5) Q20 (%) and Q30 (%): Evaluate the quality of sequencing data after quality control. Q20 and Q30 refer to the percentage of bases with sequencing quality above 99% and 99.9%, respectively, in the total number of bases. Generally, Q20 is above 85% and Q30 is above 80%; (6) GC content (%): The percentage of the total number of G and C bases corresponding to the quality control data to the total number of bases.

**Table S5 Summary of Sequence Comparison Results**

| Sample | Total reads | Total mapped | Multiple mapped | Uniquely mapped |
| --- | --- | --- | --- | --- |
| Nm_0_1 | 48683278 | 45001714 (92.44%) | 1195245 (2.46%) | 43806469 (89.98%) |
| Nm_0_2 | 48603926 | 43114023 (88.7%) | 1385606 (2.85%) | 41728417 (85.85%) |
| Nm_0_3 | 42700086 | 40009610 (93.7%) | 1140082 (2.67%) | 38869528 (91.03%) |
| Wm_0_1 | 42331688 | 40024887 (94.55%) | 1151392 (2.72%) | 38873495 (91.83%) |
| Wm_0_2 | 42285872 | 40019397 (94.64%) | 1171057 (2.77%) | 38848340 (91.87%) |
| Wm_0_3 | 42351066 | 40005014 (94.46%) | 1148839 (2.71%) | 38856175 (91.75%) |
| Wm_30_1 | 45238624 | 43048322 (95.16%) | 1331647 (2.94%) | 41716675 (92.21%) |
| Wm_30_2 | 42741628 | 40746421 (95.33%) | 1266301 (2.96%) | 39480120 (92.37%) |
| Wm_30_3 | 42348118 | 40386350 (95.37%) | 1242919 (2.94%) | 39143431 (92.43%) |
| Wm_90_1 | 43187514 | 41025156 (94.99%) | 1120122 (2.59%) | 39905034 (92.4%) |
| Wm_90_2 | 41133398 | 39162563 (95.21%) | 1082655 (2.63%) | 38079908 (92.58%) |
| Wm_90_3 | 48850100 | 47087545 (96.39%) | 1311308 (2.68%) | 45776237 (93.71%) |
| Nm_30_1 | 42745584 | 40427846 (94.58%) | 1189482 (2.78%) | 39238364 (91.8%) |
| Nm_30_2 | 51098366 | 48435930 (94.79%) | 1481228 (2.9%) | 46954702 (91.89%) |
| Nm_30_3 | 51778412 | 49115691 (94.86%) | 1500398 (2.9%) | 47615293 (91.96%) |
| Nm_90_1 | 45664770 | 42698360 (93.5%) | 1257523 (2.75%) | 41440837 (90.75%) |
| Nm_90_2 | 48279664 | 44466318 (92.1%) | 1339058 (2.77%) | 43127260 (89.33%) |
| Nm_90_3 | 49861600 | 46601195 (93.46%) | 1377219 (2.76%) | 45223976 (90.7%) |
| Ax_0_1 | 45097412 | 43000666 (95.35%) | 1327257 (2.94%) | 41673409 (92.41%) |
| Ax_0_2 | 48730876 | 46374156 (95.16%) | 1425332 (2.92%) | 44948824 (92.24%) |
| Ax_0_3 | 42366030 | 40384269 (95.32%) | 1240469 (2.93%) | 39143800 (92.39%) |
| Ax_30_1 | 47524262 | 44704908 (94.07%) | 1364679 (2.87%) | 43340229 (91.2%) |
| Ax_30_2 | 44803974 | 41757042 (93.2%) | 1289744 (2.88%) | 40467298 (90.32%) |
| Ax_30_3 | 48893472 | 45860525 (93.8%) | 1449443 (2.96%) | 44411082 (90.83%) |
| Ax_90_1 | 41991328 | 39981788 (95.21%) | 1188570 (2.83%) | 38793218 (92.38%) |
| Ax_90_2 | 56933816 | 54085571 (95.0%) | 1685595 (2.96%) | 52399976 (92.04%) |
| Ax_90_3 | 44259842 | 42065020 (95.04%) | 1274590 (2.88%) | 40790430 (92.16%) |

Note: (1) Sample: Sample name; (2) Total reads: Statistics of the number of filtered sequencing sequences (i.e. Clean reads); (3) Total mapped: The number of Clean reads that can be located on the genome; (4) Multiple mapped: The number of Clean reads with multiple aligned positions on the reference sequence; (5) Unique mapped: The number of Clean reads with unique alignment positions on the reference sequence.

**Table S6 The effect of soil phosphorus availability on soybean root morphology**

| Variety | P treatment | average diameter  (mm) | Root density  (g·cm^-3^) | Root/Shoot ratio |
| --- | --- | --- | --- | --- |
| Qd11 | P0 | 0.423±0.009b | 0.107±0.002b | 0.460±0.021a |
|  | P30 | 0.437±0.015b | 0.121±0.008b | 0.187±0.009b |
|  | P60 | 0.46±0.017b | 0.173±0.009a | 0.140±0.020b |
|  | P90 | 0.533±0.033a | 0.13±0.012b | 0.150±0.006b |
|  | P120 | 0.553±0.009a | 0.111±0.002b | 0.157±0.003b |
| Zh13 | P0 | 0.43±0.006b | 0.118±0.002c | 0.360±0.0260a |
|  | P30 | 0.493±0.015a | 0.151±0.010b | 0.193±0.012b |
|  | P60 | 0.487±0.013ab | 0.158±0.006b | 0.153±0.009b |
|  | P90 | 0.487±0.023ab | 0.168±0.014b | 0.187±0.009b |
|  | P120 | 0.5±0.025a | 0.209±0.007a | 0.180±0.010b |
| Ax | P0 | 0.437±0.023b | 0.113±0.007b | 0.440±0.079a |
|  | P30 | 0.51±0.026ab | 0.126±0.010ab | 0.233±0.027b |
|  | P60 | 0.537±0.026a | 0.146±0.013a | 0.217±0.026b |
|  | P90 | 0.537±0.028a | 0.144±0.008a | 0.153±0.018b |
|  | P120 | 0.46±0.02ab | 0.148±0.003a | 0.110±0.015b |
| Nm | P0 | 0.447±0.023b | 0.080±0.009c | 0.500±0.031a |
|  | P30 | 0.49±0.021ab | 0.089±0.003c | 0.173±0.003c |
|  | P60 | 0.523±0.029a | 0.123±0.005b | 0.263±0.007b |
|  | P90 | 0.513±0.019ab | 0.143±0.001a | 0.207±0.009c |
|  | P120 | 0.493±0.009ab | 0.141±0.007ab | 0.180±0.010c |
| Wm82 | P0 | 0.41±0.026b | 0.092±0.010c | 0.290±0.031a |
|  | P30 | 0.46±0.026b | 0.147±0.012b | 0.240±0.026ab |
|  | P60 | 0.433±0.007b | 0.194±0.009a | 0.177±0.017b |
|  | P90 | 0.553±0.035a | 0.133±0.006b | 0.173±0.013b |
|  | P120 | 0.487±0.033ab | 0.135±0.009b | 0.187±0.032b |

Note: Different lowercase letters within a column indicate significant differences among P levels for a given variety (Duncan, P < 0.05)

**Table S7 The effect of soil phosphorus availability on soybean root exudates**

| Variety | P treatment | Oxalic acid  mg·h^-1^·g^-1^ | Acetic acid  mg·h^-1^·g^-1^ | Citric acid  mg·h^-1^·g^-1^ | Succinic acid  mg·h^-1^·g^-1^ | Malic acid  mg·h^-1^·g^-1^ | Tartaric acid  mg·h^-1^·g^-1^ |
| --- | --- | --- | --- | --- | --- | --- | --- |
| Qd11 | P0 | 8.592±0.422a | 1.805±0.084b | 0.508±0.030a | 0.257±0.014b | 0.031±0.002b | 8.186±0.662a |
|  | P30 | 2.104±0.127b | 3.698±0.143a | 0.293±0.033b | 0.640±0.078a | 0.062±0.004a | 2.431±0.139b |
|  | P60 | 0.320±0.019c | 1.035±0.075c | 0.191±0.017c | 0.079±0.001c | 0.014±0.001c | 0.833±0.040c |
|  | P90 | 0.110±0.022c | 0.803±0.075c | 0.041±0.001d | 0.19±0.016bc | 0.015±0.002c | 0.283±0.015c |
|  | P120 | 0.127±0.014c | 0.096±0.005d | 0.047±0.003d | 0.166±0.013bc | 0.012±0.002c | 0.246±0.012c |
| Zh13 | P0 | 7.840±0.488a | 4.964±0.291a | 0.271±0.011a | 0.426±0.037a | 0.056±0.002a | 8.356±0.436a |
|  | P30 | 1.139±0.035b | 1.853±0.075b | 0.191±0.015b | 0.282±0.019b | 0.024±0.002b | 1.396±0.042b |
|  | P60 | 0.231±0.014c | 0.494±0.039c | 0.162±0.012b | 0.206±0.011c | 0.022±0.002b | 0.577±0.029c |
|  | P90 | 0.159±0.017c | 0.962±0.050d | 0.091±0.001c | 0.126±0.003d | 0.012±0.001c | 0.525±0.037c |
|  | P120 | 0.143±0.007c | 0.107±0.007d | 0.092±0.008c | 0.028±0.004e | 0.020±0.001b | 0.245±0.017c |
| Ax | P0 | 11.051±1.213a | 1.150±0.102a | 0.436±0.053a | 0.246±0.017a | 0.093±0.017a | 9.900±1.026a |
|  | P30 | 1.942±0.156b | 0.797±0.056b | 0.245±0.017b | 0.154±0.006b | 0.041±0.003b | 2.38±0.168b |
|  | P60 | 0.144±0.008c | 0.212±0.014c | 0.126±0.002c | 0.047±0.003d | 0.014±0.001c | 0.338±0.013c |
|  | P90 | 0.070±0.003c | 0.189±0.012c | 0.016±0.001cd | 0.100±0.003c | 0.011±0.001c | 0.186±0.007c |
|  | P120 | 0.086±0.005c | 0.105±0.006c | 0.086±0.017d | 0.116±0.008c | 0.016±0.002c | 0.264±0.012c |
| Nm | P0 | 5.935±0.520a | 1.095±0.086b | 0.191±0.017a | 0.172±0.012b | 0.032±0.003a | 4.623±0.305a |
|  | P30 | 0.776±0.008b | 1.454±0.132a | 0.203±0.011a | 0.329±0.021a | 0.025±0.000ab | 1.202±0.018b |
|  | P60 | 0.134±0.006b | 0.217±0.022c | 0.117±0.016b | 0.090±0.006c | 0.023±0.007ab | 0.311±0.008c |
|  | P90 | 0.038±0.002b | 0.054±0.007c | 0.069±0.004d | 0.109±0.003c | 0.022±0.003ab | 0.075±0.005c |
|  | P120 | 0.044±0.003b | 0.063±0.005c | 0.114±0.010c | 0.102±0.018c | 0.014±0.002b | 0.087±0.006c |
| Wm82 | P0 | 7.632±0.605a | 1.498±0.048a | 0.216±0.020a | 0.257±0.010a | 0.047±0.007a | 7.278±0.539a |
|  | P30 | 1.233±0.154b | 0.498±0.011b | 0.133±0.016b | 0.197±0.013b | 0.028±0.002b | 1.292±0.04b |
|  | P60 | 0.204±0.012c | 0.325±0.029c | 0.091±0.015bc | 0.082±0.003d | 0.013±0.000c | 0.405±0.015c |
|  | P90 | 0.180±0.007c | 0.257±0.013c | 0.072±0.009cd | 0.111±0.010cd | 0.010±0.000c | 0.350±0.014c |
|  | P120 | 0.168±0.012c | 0.319±0.012c | 0.037±0.001d | 0.135±0.009d | 0.011±0.001c | 0.409±0.005c |

**Table S8 Expression of important genes in soybean response to phosphorus starvation**

| Gene family | | Gene ID | Gene Name | | Gene description | Nm | | Wm82 | | Ax | |
| --- | --- | --- | --- | --- | --- | --- | --- | --- | --- | --- | --- |
|  |  |  |  |  |  | FC | Padjust | FC | Padjust n | FC | Padjust |
| P0 vs P90 | | | | | | | | | | | |
| PHT | | Glyma.10G186400 | GmPHT1;6 | | phosphate transporter 1-6 | 32.803 | 2.71833E-32 | 18.249 | 1.49926E-32 | 6.246 | 5.76546E-21 |
|  |  | Glyma.10G186500 | GmPHT1;7 | | phosphate transporter 1-7 | 7.418 | 1.0896E-129 | 4.635 | 5.2038E-258 | 6.491 | 0 |
|  |  | Glyma.20G204100 | GmPHT1;14 | | phosphate transporter 1-14 | 710.165 | 1.81009E-26 | 377.557 | 7.96868E-12 | 48.261 | 4.85737E-51 |
|  |  | Glyma.02G005800 | GmPHT1;1 | | phosphate transporter 1-1 | 28.111 | 1.0034E-249 | 11.422 | 0 | 27.143 | 0 |
|  |  | Glyma.14G123500 | GmPHT1;9 | | phosphate transporter 1-9 | 1.173 | 0.892119975726 | 3.935 | 0.000288610249973 | 58.011 | 7.14915580266E-19 |
|  |  | Glyma.13G040200 | GmPHT1;8 | | phosphate transporter 1-8 | 68.6889358193 | 1.21241104493E-5 | 19.6485437859 | 7.71123250296E-16 | 188.751426141 | 1.34602090245E-17 |
|  |  | Glyma.19G164300 | GmPHT1;11 | | phosphate transporter 1-11 | 22.636 | 0 | 13.671 | 0 | 14.572 | 0 |
|  |  | Glyma.10G006700 | GmPHT1;4 | | phosphate transporter 1-4 | 12.972 | 1.61946E-87 | 16.554 | 0 | 17.331 | 0 |
| PHO | | Glyma.01G091800 | GmPHO1;H12 | | phosphate transporter PHO1 homolog 1 | 16.933 | 4.49036E-17 | 2.569 | 4.73518E-05 | 6.828 | 6.96313E-27 |
|  |  | Glyma.02G003700 | Glyma.02G003700 | | phosphate transporter PHO1 | 2.712 | 3.41586E-06 | 1.644 | 1.54958E-09 | 4.795 | 1.9943E-118 |
|  |  | Glyma.02G130200 | Glyma.02G130200 | | phosphate transporter PHO1 | 10.389 | 5.09505E-30 | 2.697 | 1.13986E-13 | 5.813 | 4.90666E-53 |
| PAP | | Glyma.05G138400 | Glyma.05G138400 | | purple acid phosphatase 8-like precursor | 19.761 | 3.7006E-102 | 18.525 | 0 | 24.345 | 0 |
|  |  | Glyma.05G247800 | Glyma.05G247800 | | purple acid phosphatase 3-like precursor | 53.293 | 1.66114E-82 | 4.725 | 1.4355E-104 | 12.711 | 0 |
|  |  | Glyma.08G056400 | Glyma.08G056400 | | purple acid phosphatase 17 isoform X1 | 58.211 | 4.0687E-283 | 93.934 | 0 | 124.945 | 1.1186E-146 |
|  |  | Glyma.08G093600 | GmPAP7 | | purple acid phosphatase 7 | 26.265 | 1.254E-117 | 17.559 | 0 | 15.909 | 2.15838E-80 |
|  |  | Glyma.10G071000 | Glyma.10G071000 | | purple acid phosphatase 22-like | 22.107 | 1.94468E-84 | 28.462 | 0 | 31.242 | 0 |
| PHR | | Glyma.03G143600 | GmPHR8 | | MYB-CC domain-containing transcription factor PHR8 | 9.756 | 1.36781E-16 | 5.590 | 8.45854E-34 | 6.303 | 8.53656E-34 |
|  |  | Glyma.10G039700 | GmPHR17 | | MYB-CC domain-containing transcription factor PHR17 | 9.475 | 4.19826E-26 | 2.897 | 2.02548E-19 | 7.399 | 6.76769E-65 |
|  |  | Glyma.12G089100 | GmPHR20 | | MYB-CC domain-containing transcription factor PHR20 | 2.096 | 0.006047071 | 1.474 | 0.00051873 | 1.952  3 | 9.67455E-15 |
|  |  | Glyma.13G126200 | GmPHR22 | | MYB-CC domain-containing transcription factor PHR22 | 3.322 | 1.66203E-14 | 1.206 | 0.03630446 | 1.815 | 6.72447E-14 |
|  |  | Glyma.15G123100 | GmPHR25 | | MYB-CC domain-containing transcription factor PHR25 | 3.013 | 3.28464E-44 | 1.592 | 3.95793E-15 | 1.542 | 4.3926E-19 |
|  |  | Glyma.18G201800 | GmPHR29 | | MYB-CC domain-containing transcription factor PHR29 | 18.653 | 9.43884E-07 | 20.156 | 4.601E-203 | 10.186 | 8.30441E-62 |
|  |  | Glyma.19G146600 | GmPHR31 | | MYB-CC domain-containing transcription factor PHR31 | 2.659 | 0.002189137 | 4.671 | 4.19243E-27 | 8.343 | 7.39044E-74 |
| ALMT | | Glyma.05G233400 | ALMT9 | | aluminum-activated malate transporter 9 | 3.411 | 2.35927E-11 | 5.166 | 2.11961E-18 | 1.906 | 0.001059126 |
|  |  | Glyma.19G199900 | ALMT8 | | aluminum-activated malate transporter 8 | 1.192 | 0.907027979 | 14.037 | 0.000617432 | 19.846 | 0.048828821 |
| Gene family | | Gene ID | Gene Name | | Gene description | Nm | | Wm82 | | Ax | |
|  |  |  |  |  |  | FC | Padjust | FC | Padjust | FC | Padjust |
| P30 vs P90 | | | | | | | | | | | |
| PHT | Glyma.10G186400 | | GmPHT1;6 | phosphate transporter 1-6 | | 8.292 | 3.31246E-10 | 4.349 | 2.65633E-05 | 3.613 | 1.0953E-08 |
|  | Glyma.10G186500 | | GmPHT1;7 | phosphate transporter 1-7 | | 2.241 | 1.49945E-58 | 2.926 | 2.4045E-142 | 4.259 | 2.8792E-262 |
|  | Glyma.20G204100 | | GmPHT1;14 | phosphate transporter 1-14 | | 40.808 | 6.86264E-08 | 97.038 | 1.18193E-06 | 36.628 | 2.23792E-40 |
|  | Glyma.02G005800 | | GmPHT1;1 | phosphate transporter 1-1 | | 17.427 | 0 | 3.178 | 1.7069E-154 | 32.691 | 0 |
|  | Glyma.14G123500 | | GmPHT1;9 | phosphate transporter 1-9 | | 13.984 | 1.76701015645E-13 | 6.856 | 1.52964225862E-10 | 109.736 | 1.0049938E-26 |
|  | Glyma.13G040200 | | GmPHT1;8 | phosphate transporter 1-8 | | 85.779 | 2.39494593148E-8 | 10.065 | 2.2789260549E-8 | 160.942 | 3.5797549E-16 |
|  | Glyma.19G164300 | | GmPHT1;11 | phosphate transporter 1-11 | | 4.524 | 0 | 8.012 | 0 | 11.168 | 0 |
|  | Glyma.10G006700 | | GmPHT1;4 | phosphate transporter 1-4 | | 15.079 | 0 | 5.025 | 0 | 32.777 | 0 |
| PHO | Glyma.01G091800 | | GmPHO1;H12 | phosphate transporter PHO1 homolog 1 | | 4.475 | 6.91033E-05 | 2.429 | 2.6409E-05 | 5.056 | 1.6102E-16 |
|  | Glyma.02G003700 | | Glyma.02G003700 | phosphate transporter PHO1 | | 1.021 | 0.926173085 | 0.747 | 0.004455989 | 2.551 | 2.34328E-30 |
|  | Glyma.02G130200 | | Glyma.02G130200 | phosphate transporter PHO1 | | 4.104 | 2.19504E-14 | 3.027 | 9.02288E-21 | 3.320 | 4.2994E-22 |
| PAP | Glyma.05G138400 | | Glyma.05G138400 | purple acid phosphatase 8-like precursor | | 6.129 | 7.3988E-151 | 10.468 | 5.5077E-192 | 19.789 | 5.5699E-252 |
|  | Glyma.05G247800 | | Glyma.05G247800 | purple acid phosphatase 3-like precursor | | 11.699 | 1.4408E-107 | 2.844 | 0 | 10.861 | 1.47314E-44 |
|  | Glyma.08G056400 | | Glyma.08G056400 | purple acid phosphatase 17 isoform X1 | | 19.316 | 0 | 69.986 | 0 | 100.533 | 8.5925E-137 |
|  | Glyma.08G093600 | | GmPAP7 | purple acid phosphatase 7 | | 10.226 | 2.7804E-256 | 8.683 | 6.4449E-199 | 14.907 | 2.47192E-73 |
|  | Glyma.10G071000 | | Glyma.10G071000 | purple acid phosphatase 22-like | | 11.061 | 2.5103E-188 | 5.927 | 5.786E-114 | 28.261 | 0 |
| PHR | Glyma.03G143600 | | GmPHR8 | MYB-CC domain-containing transcription factor PHR8 | | 1.209 | 0.721497196 | 0.876 | 0.676134843 | 0.929 | 0.8644695 |
|  | Glyma.10G039700 | | GmPHR17 | MYB-CC domain-containing transcription factor PHR17 | | 2.691 | 1.89799E-13 | 2.492 | 9.31764E-14 | 5.772 | 3.79626E-47 |
|  | Glyma.12G089100 | | GmPHR20 | MYB-CC domain-containing transcription factor PHR20 | | 2.119 | 1.63944E-24 | 2.210 | 3.76482E-18 | 1.431 | 0.000366478 |
|  | Glyma.13G126200 | | GmPHR22 | MYB-CC domain-containing transcription factor PHR22 | | 1.149 | 0.253805232 | 0.892 | 0.260620987 | 1.542 | 3.81632E-07 |
|  | Glyma.15G123100 | | GmPHR25 | MYB-CC domain-containing transcription factor PHR25 | | 1.092 | 0.235866091 | 0.933 | 0.386490047 | 1.293 | 1.03219E-05 |
|  | Glyma.18G201800 | | GmPHR29 | MYB-CC domain-containing transcription factor PHR29 | | 5.375 | 3.61841E-90 | 4.429 | 8.47016E-27 | 15.351 | 1.44993E-92 |
|  | Glyma.19G146600 | | GmPHR31 | MYB-CC domain-containing transcription factor PHR31 | | 0.743 | 0.333703435 | 1.217 | 0.449496054 | 1.201 | 0.521916038 |
| ALMT | Glyma.05G233400 | | ALMT9 | aluminum-activated malate transporter 9 | | 0.877 | 0.675724959 | 2.505 | 5.87187E-05 | 1.098 | 0.791936977 |
|  | Glyma.19G199900 | | ALMT8 | aluminum-activated malate transporter 8 | | 0.071 | 0.143796136 | 1.208 | 0.93851603 | 4.061 | 1 |
