# Supplementary material for: Phosphorus-dependent shifts in acquisition strategies revealed by integrated transcriptomics and metabolomics in soybean roots
Source: BMC Plant Biol. 2025 Dec 17;25:1753. doi: 10.1186/s12870-025-07957-x (PMC12751778; doi:10.1186/s12870-025-07957-x)
Supplement: Supplementary file 2 — Supplementary Material 2. Figure S1. Effects of P availability on soybean growth. Plant height and stem diameter under different P treatments. Lowercase letters indicate significant differences among treatments within the same cultivar (P < 0.05). Asterisks denote significance at *P < 0.05 and **P < 0.01. Figure S2. KEGG enrichment of differential metabolites. KEGG pathway enrichment of differentially accumulated metabolites (DAMs) in soybean roots under P treatments. Figure S3. KEGG enrichment of differentially expressed genes. (A) Expression profiles of root DEGs under different P treatments. (B) PCA of gene expression in three soybean cultivars. (C) KEGG pathway enrichment of DEGs in soybean roots. [file 12870_2025_7957_MOESM2_ESM.docx]

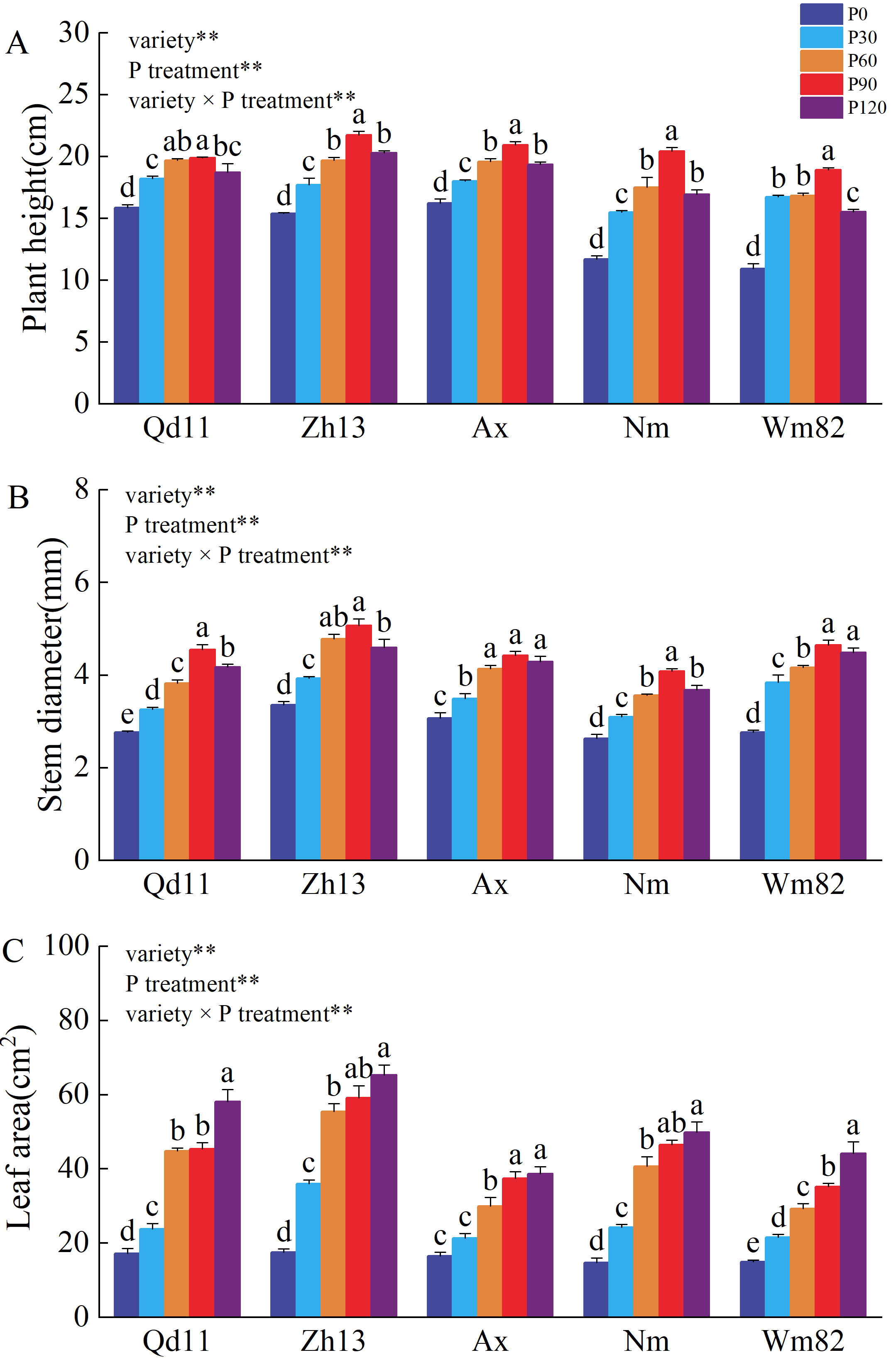


Figure S1 The effects of soil phosphorus availability on plant height and stem diameter of soybean

Note: Lowercase letters indicate significant differences in the same variety under different phosphorus treatments (*P* < 0.05), and "*, **" represent significance at the 0.05 and 0.01 levels, respectively. This convention applies to all supplementary figures unless otherwise stated.


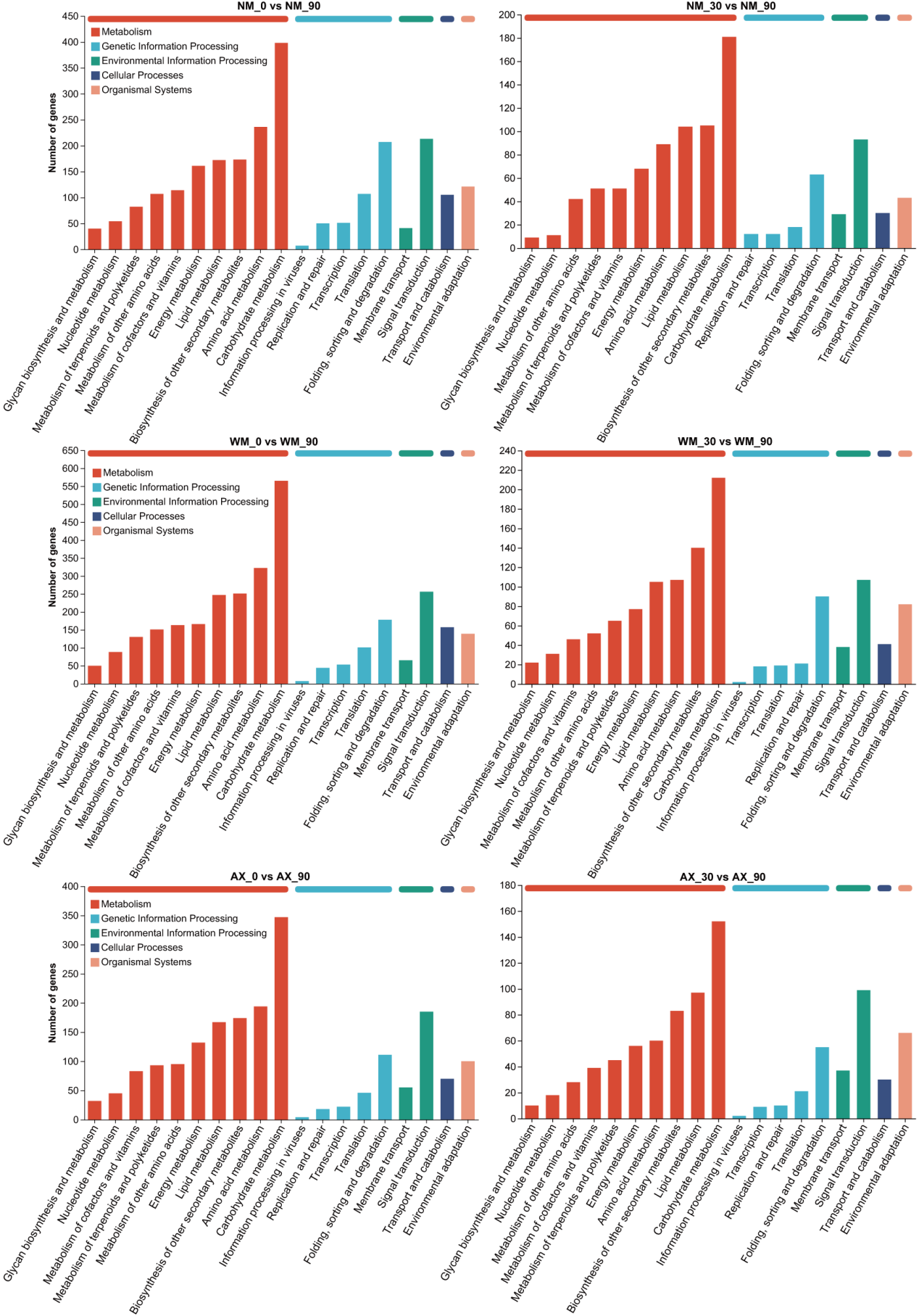


Figure S2 KEGG enrichment analysis of DAMs in soybean roots


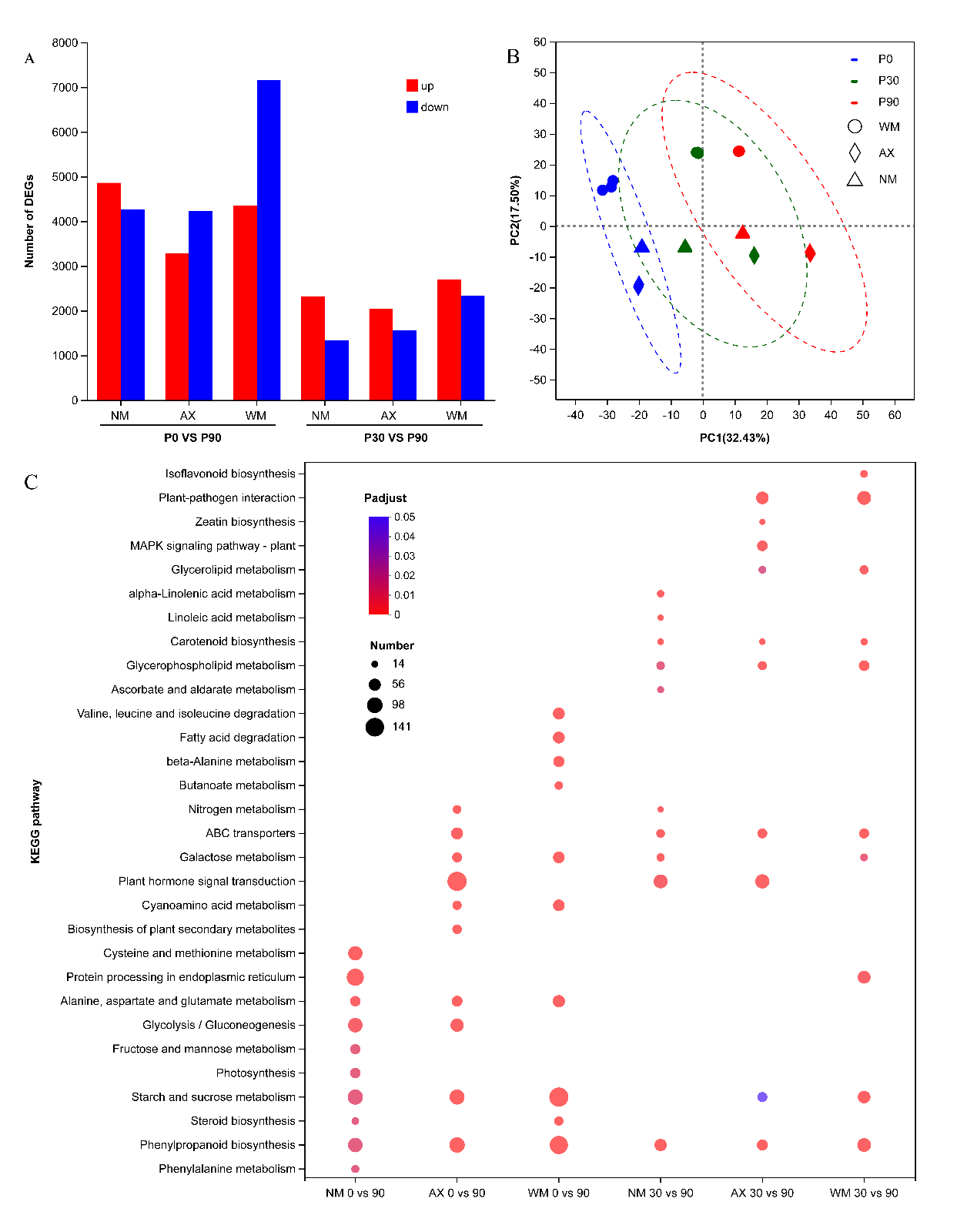


Figure S3 KEGG enrichment analysis of differentially expressed genes in soybean root system

Note: A, represents the expression of root DEGs under different phosphorus treatments; B, PCA analysis of gene expression in three soybean varieties under different phosphorus treatments; C, KEGG enrichment analysis of differentially expressed genes in soybean root system.
